# Galectin-4 N-Terminal Domain: Binding Preferences Toward A and B Antigens With Different Peripheral Core Presentations

**DOI:** 10.3389/fchem.2021.664097

**Published:** 2021-04-21

**Authors:** Jon I. Quintana, Sandra Delgado, Reyes Núñez-Franco, F. Javier Cañada, Gonzalo Jiménez-Osés, Jesús Jiménez-Barbero, Ana Ardá

**Affiliations:** ^1^CIC bioGUNE, Basque Research and Technology Alliance (BRTA), Derio, Spain; ^2^Margarita Salas Center for Biological Research, Centro de Investigaciones Biológicas Margarita Salas, Spanish National Research Council, Madrid, Spain; ^3^CIBER de Enfermedades Respiratorias (CIBERES) Avda, Monforte de Lemos, Spain; ^4^lkerbasque, Basque Foundation for Science, Bilbao, Spain; ^5^Department of Organic Chemistry ll, Faculty of Science & Technology, University of the Basque Country, Leioa, Spain

**Keywords:** NMR, molecular recognition, galectin-4, blood type antigen, lectin—carbohydrate interaction

## Abstract

The tandem-repeat Galectin-4 (Gal-4) contains two different domains covalently linked through a short flexible peptide. Both domains have been shown to bind preferentially to A and B histo blood group antigens with different affinities, although the binding details are not yet available. The biological relevance of these associations is unknown, although it could be related to its attributed role in pathogen recognition. The presentation of A and B histo blood group antigens in terms of peripheral core structures differs among tissues and from that of the antigen-mimicking structures produced by pathogens. Herein, the binding of the N-terminal domain of Gal-4 toward a group of differently presented A and B oligosaccharide antigens in solution has been studied through a combination of NMR, isothermal titration calorimetry (ITC), and molecular modeling. The data presented in this paper allow the identification of the specific effects that subtle chemical modifications within this antigenic family have in the binding to the N-terminal domain of Gal-4 in terms of affinity and intermolecular interactions, providing a structural-based rationale for the observed trend in the binding preferences.

## Introduction

Galectin-4 (Gal-4) is one of the 16 members of the human galectin family, characterized by their participation in a myriad of biological phenomena, with important implications in immunity (Sato et al., 2009; Giovannone et al., 2018; Martínez Allo et al., 2018), inflammation, and cancer (Elola et al., 2018). They exert their functions through their ability to bind β-galactoside-containing glycans, toward which the different galectins present different affinities and selectivities.

The physiological roles of Gal-4 have been reviewed (Cao and Guo, 2016) and include apical protein trafficking, intestinal wound healing, lipid raft stabilization, and bacterial pathogen fighting. In healthy individuals, Gal-4 is predominantly expressed in the epithelial cells along the alimentary tract; although, similar to other members of the galectin family, its distribution in cancer is altered (Huflejt and Leffler, 2003). Although its exact roles in the disease are not fully understood, the correlation between Gal-4 levels and malignancy has been reported for many types of cancer (Tsai et al., 2016). These pieces of evidence have in fact fostered the use of Galectins as targets for therapeutic intervention for several types of cancer (Cagnoni et al., 2016; Girard and Magnani, 2018).

Galectin-4 belongs to the tandem-repeat galectins subfamily, characterized by displaying two different carbohydrate recognition domains (CRDs) covalently linked through a short peptide. The role of this peptide linker in the overall protein behavior is not yet clear, neither is its influence on the carbohydrate-binding properties. In the case of Gal-4, the two CRDs show a 49% similarity and, despite sharing certain glycan-binding preferences, they do not completely overlap. Previous studies on the binding preferences of the full-length form of Gal-4 (Ideo et al., 2002, 2005; Vokhmyanina et al., 2012) as well as on the two independent N- and C-terminal domains (Huflejt and Leffler, 2003) have revealed their preference toward the human histo blood group antigens (HBGAs) A and B. Despite certain controversy (Vokhmyanina et al., 2012), most studies agree on the fact that the N-terminal domain of Gal-4 (Gal-4N) recognizes A and B blood group antigens with less affinity than the C-terminal domain (Gal-4C). Nevertheless, significant discrepancies on the binding affinities were reported (Ideo et al., 2005, 2011; Bum-Erdene et al., 2016). X-ray crystallography analysis has provided structural details on how the two independent CRDs recognize different linear lactose derivatives, including 2′-fucosyl lactose (H type-6 antigen) (Bum-Erdene et al., 2015, 2016). However, no experimental data are yet available for the recognition of branched antigens, including the blood-type antigens.

**Scheme 1:**
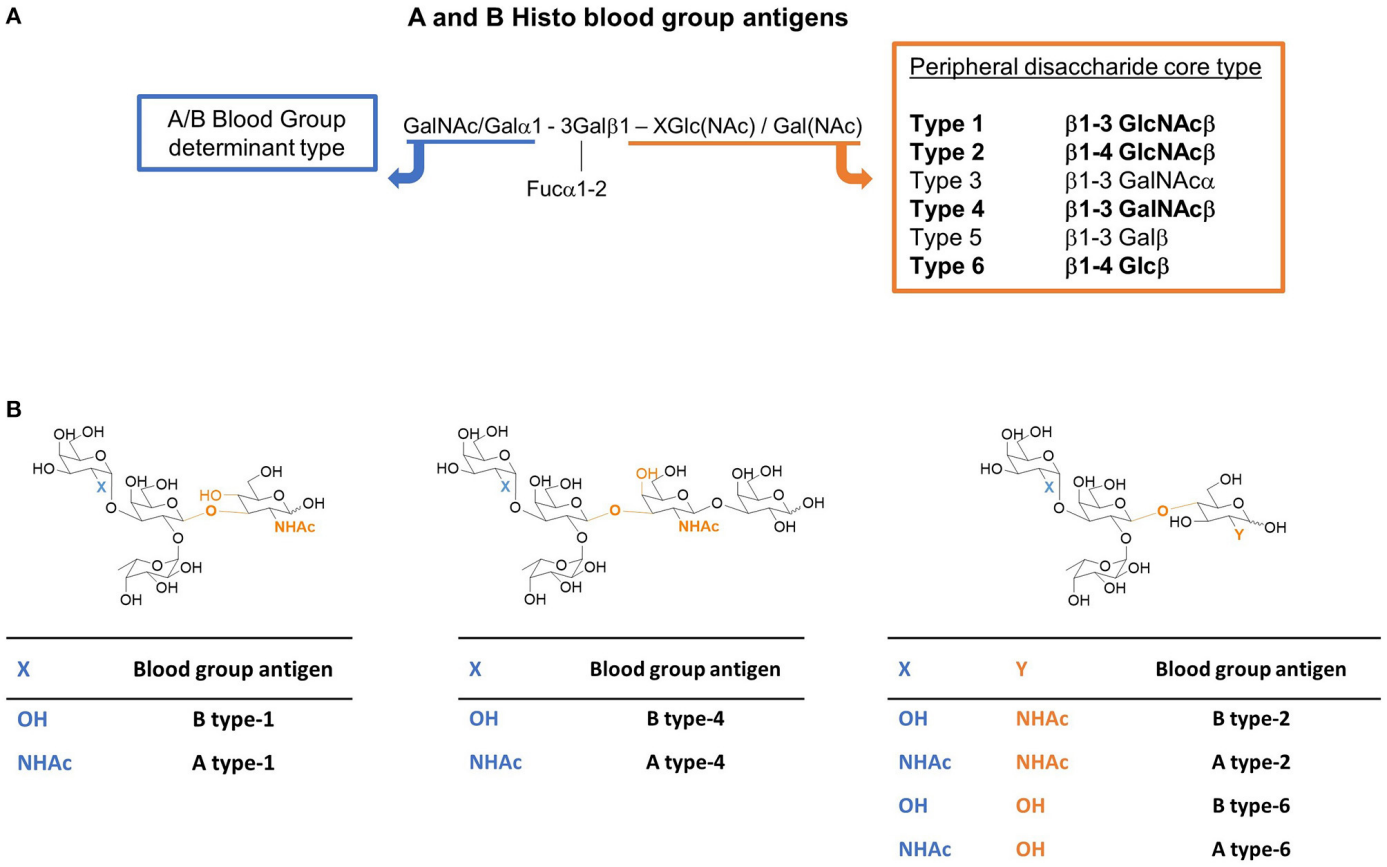
**(A)** The A and B histo blood group antigens and their possible peripheral disaccharide core structures (the core types studied herein are in bold). X stands for the linked atom at the reducing-end residue (X = 3 for types-1 and -4; X = 4 for types-2 and -6). **(B)** Tetra- and pentasaccharide representative structures of the A and B blood group antigens of type-1, -2, -4, and -6 studied herein. The difference between A and B antigens is the substituent at C2 of the terminal residue (highlighted in blue): -OH (Gal) for the B antigen, and NHAc (GalNAc) for the A antigen. The type depends on the peripheral disaccharide core structure whose structural differences are highlighted in orange.

In particular, the ABO antigens are divided into six groups, depending on their peripheral core disaccharide structures (Scheme 1), namely type-1 (Galβ1-3GlcNAcβ), type-2 (Galβ1-4GlcNAcβ), type-3 (Galβ1-3GalNAcβ), type-4 (Galβ1-3GalNAcβ), type-5 (Galβ1-3Glcβ), and type-6 (Galβ1-4Glcβ), giving rise to a family of epitopes which are presented in different manners. This different presentation is known to influence their antigenicity (Watkins et al., 1988; Clausen and Hakomori, 1989; Tanaka et al., 2008). Moreover, their distribution is also different. HBGAs are mostly present as type-2 structures (N-acetyl lactosamine, Galβ1-4GlcNAcβ). However, HBGAs are not only found on red blood cells but also on the cell surfaces of most endothelial and epithelial cells and in the secretions of the so-called AB-secretor genotype individuals. In fact, type-1 antigens are found on endodermal tissues and type-2 antigens are found on both ecto- and endo-dermal tissues, while type-3 and-4 antigens are present on ecto- or endodermally derived tissues, including the salivary glands and kidneys. While the presence of type-5 antigens in humans is controversial (Meloncelli and Lowary, 2010), type-6 antigens have been much less studied and they have been reported to be in human milk and tissue samples (Breimer et al., 1982).

From a biological perspective, the significance of the specific association of HBGAs with certain members of the galectin family seems intriguing. It has been suggested (Kamili et al., 2016) that some galectins could fill an important gap in adaptive immunity by conferring protection against pathogens that use molecular mimicry for the infection, producing blood group-like antigenic glycan structures (Arthur et al., 2015). Thus, individuals of a given blood type, who do not produce antibodies against their own blood group antigens, would then be protected by the immune activity of galectins. In support of this hypothesis, Gal-4 was demonstrated to be able to recognize and kill *Escherichia coli* bacteria expressing the blood group B antigen (Stowell et al., 2010). Interestingly, a quick glance at the O-antigenic structures produced by *E. Coli* (Stenutz et al., 2006) reveals that not only the O86 *E. Coli* serotype (that precisely express the B-antigen structure) but also other serotypes incorporate O-antigens that share common structural elements with galectin ligands. Their chemical context compared to the endogenous epitopes is however different, for instance, with respect to their neighboring residues and linkages. Considering the diversity of the ABO antigen presentation in terms of peripheral glycan structures (types), among both endogenous glycans and host-mimicking pathogenic glycans, the influence of this presentation on their recognition by Gal-4N has been explored here.

A library of commercially available HBGAs with different presentations at the peripheral disaccharide core (Scheme 1) was selected to interrogate the binding preferences of Gal-4N toward the possible chemical variations within this glycan antigenic family. In particular, this study has focused on the A vs. B preference and on the chemical variations involving their peripheral core presentation, including the glycosidic linkage (β1–3 vs. β1–4) as well as the nature of the first peripheral core monosaccharide (GlcNAc, GalNAc, or Glc). This paper presents a systematic study based on experimental NMR data and isothermal titration calorimetry (ITC) measurements, which, combined with computational chemistry tools, provide an atomic-level rationalization of the observed binding preferences of Gal-4N.

## Results and Discussion

The interaction of Gal-4N with the A and B type-1, -2, and -6 tetrasaccharides and their interaction with the A and B type-4 pentasaccharides (Scheme 1) were addressed by combining different NMR-based strategies. ^1^H-Saturation Transfer Difference (STD) NMR experiments were employed to report on the glycan binding epitope (Bhunia et al., 2012; Wagstaff et al., 2013; Marchetti et al., 2016). Additionally, ROESY experiments of ligand/Gal-4N mixtures revealed specific ligand ^1^H resonances in slow exchange in the NMR chemical shift timescale between the free and bound states. Fittingly, the analysis of their chemical shift differences (Δδ^1^H free-bound) proved to be instrumental in providing a sort of information on ligand binding epitope complementary to that obtained by using the STD experiments. Additionally, experiments from the protein perspective, based on the observation of ^15^N-labeled Gal-4N, were used to estimate binding constant affinities (K_D_) and to determine the binding epitope of the lectin in each case, which were further quantitatively measured by ITC. The synergic combination of these experimental data with molecular modeling procedures provided detailed 3D models of the complexes.

### NMR

#### ^1^H-Saturation Transfer Difference NMR

**Figure 1:**
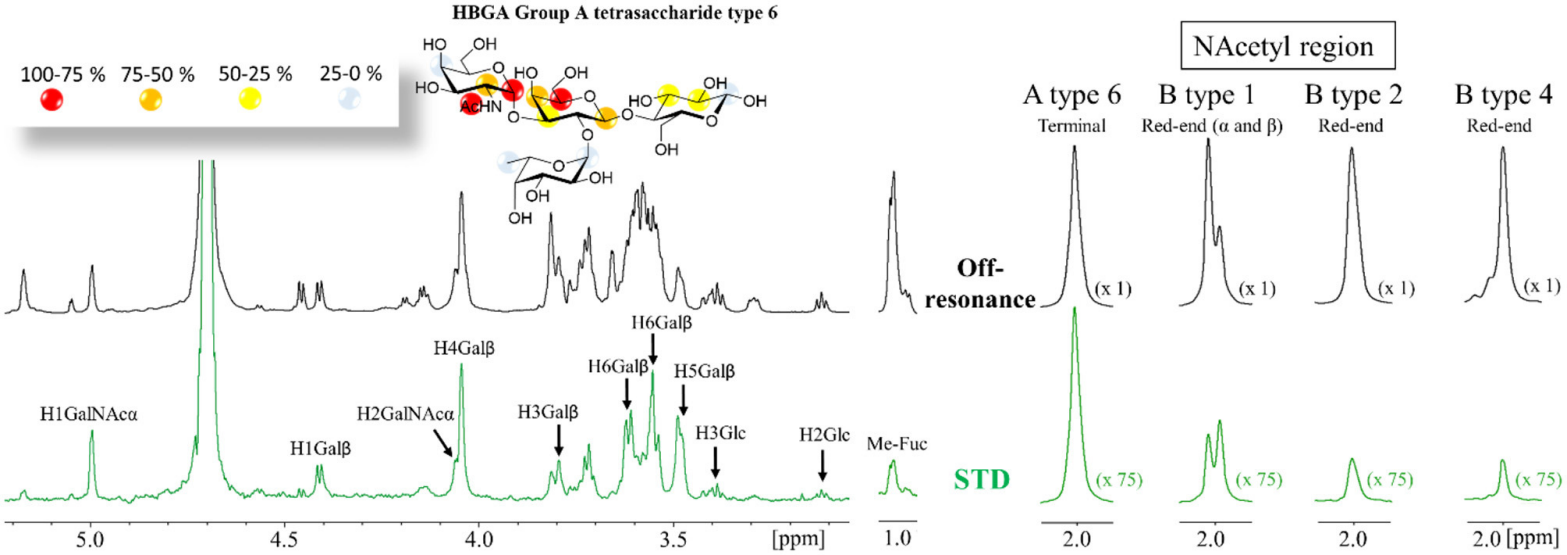
**(A)** ^1^H STD NMR experiment for the complex formed by the A type-6 tetrasaccharide and Gal-4N (50:1 molar ratio). Top: the reference spectrum (black, off-resonance). Bottom: the STD NMR spectrum (green, on-resonance at the aromatic region). The ^1^H-NMR signals showing STD effect are annotated. The epitope mapping (relative STD) is shown in the ligand structure. **(B)** Details of the off-resonance and the STD NMR spectra showing the NAc region obtained for the HBGAs in the presence of Gal-4N: from left to right: A type-6, B-type-1, B-type-2, and B-type-4. Terminal refers to the terminal α-GalNAc residue. Red-end refers to the reducing-end GlcNAc or GalNAc residues. The intensities of the STD spectra are 75-fold incremented with respect to the reference off-resonance spectra.

^1^H-saturation transfer difference NMR spectra were acquired for mixtures of Gal-4N (50 μM) with a 50-fold excess of ligand. Protein aromatic irradiation afforded the best STD intensities and thus these are discussed henceforth. The STD NMR spectra were very similar among the different ligands with respect to both the observed glycan protons and their intensities. For all the ligands, all the protons of the central βGal residue except H2, together with H1 and H2 of the terminal αGal/αGalNAc residues, showed the strongest relative STD NMR effects (above 50%) as shown in Figure 1 and Supplementary Information. Severe proton overlapping precluded a full comparison among ligands, but key isolated ^1^H NMR resonance signals permitted to highlight evident differences.

In particular, the comparison of the ^1^H-STD NMR signals originating from the acetyl groups was highly informative. For all the A-type antigens, the Ac group at the terminal GalNAc residue showed very strong STD intensity (Figure 1B). In contrast, the Ac groups at the reducing-end GlcNAc, present in type-1 (β1–3) and type-2 (β1–4) antigens, displayed weaker intensities. However, these signals were always stronger for the β1–3 than for the alternative β1–4 linked antigens (Figure 1B). This fact indicates that this NAc group is closer to protein aromatic residues (the STD on-resonance frequency irradiation) for the β1–3 linked epitopes than for the analogous NAc for β1–4 presentations.

The methyl group of the Fuc moieties showed weak STD intensities, always below 20%. Interestingly, they were systematically much weaker for the A antigens than for the B antigens (see Supplementary Information). These data clearly indicate that the Fuc residue is further away from the protein surface in the lectin complexes with the A antigens than in those with the B analogs.

**Figure 2:**
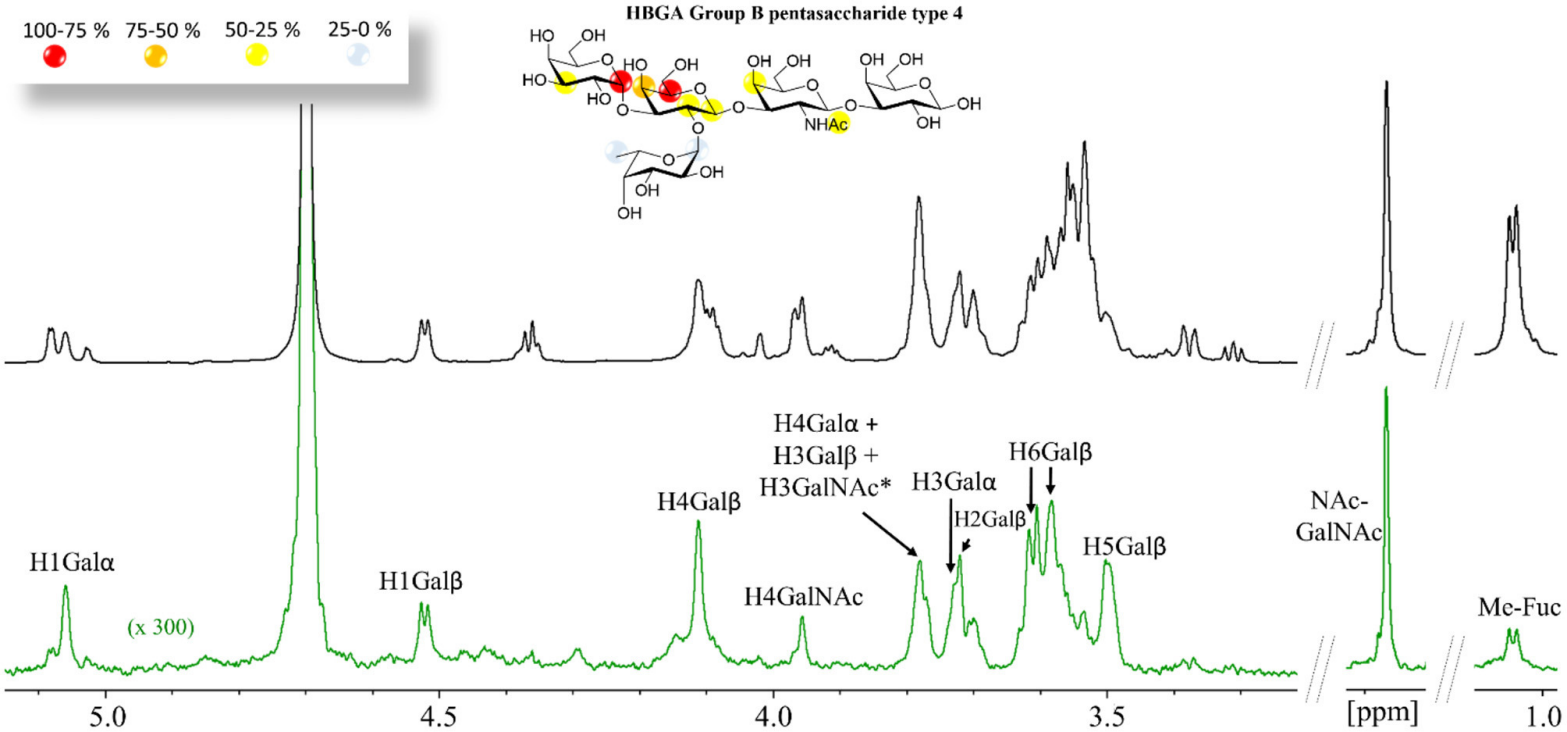
^1^H STD NMR experiment for the complex formed by the B type-4 pentasaccharide and Gal-4N (50:1 molar ratio). **Top**: the reference spectrum (black, off-resonance). **Bottom**: the STD NMR spectrum (green, on-resonance at the aromatic region). The ^1^H-NMR signals showing STD effect are annotated. The epitope mapping (relative STD) is shown in the ligand structure. *overlapping proton signals.

For the longer pentasaccharides A and B type-4 antigens, there was a remarkable lack of STD effect at the reducing-end Gal residue (Figure 2) and very weak for the βGalNAc, clearly indicating that the binding epitope implies exclusively the terminal residues. This is a fairly interesting observation since the type-4 core structure (Scheme 1) contains the GalNAcβ1-3Gal moiety at the reducing end that could act as an additional binding epitope by positioning the GalNAcβ- residue on the primary binding site (stacked on W84), increasing the apparent binding affinity. These data strongly suggest, however, that this is not the case.

#### Transverse ROESY NMR

**Figure 3:**
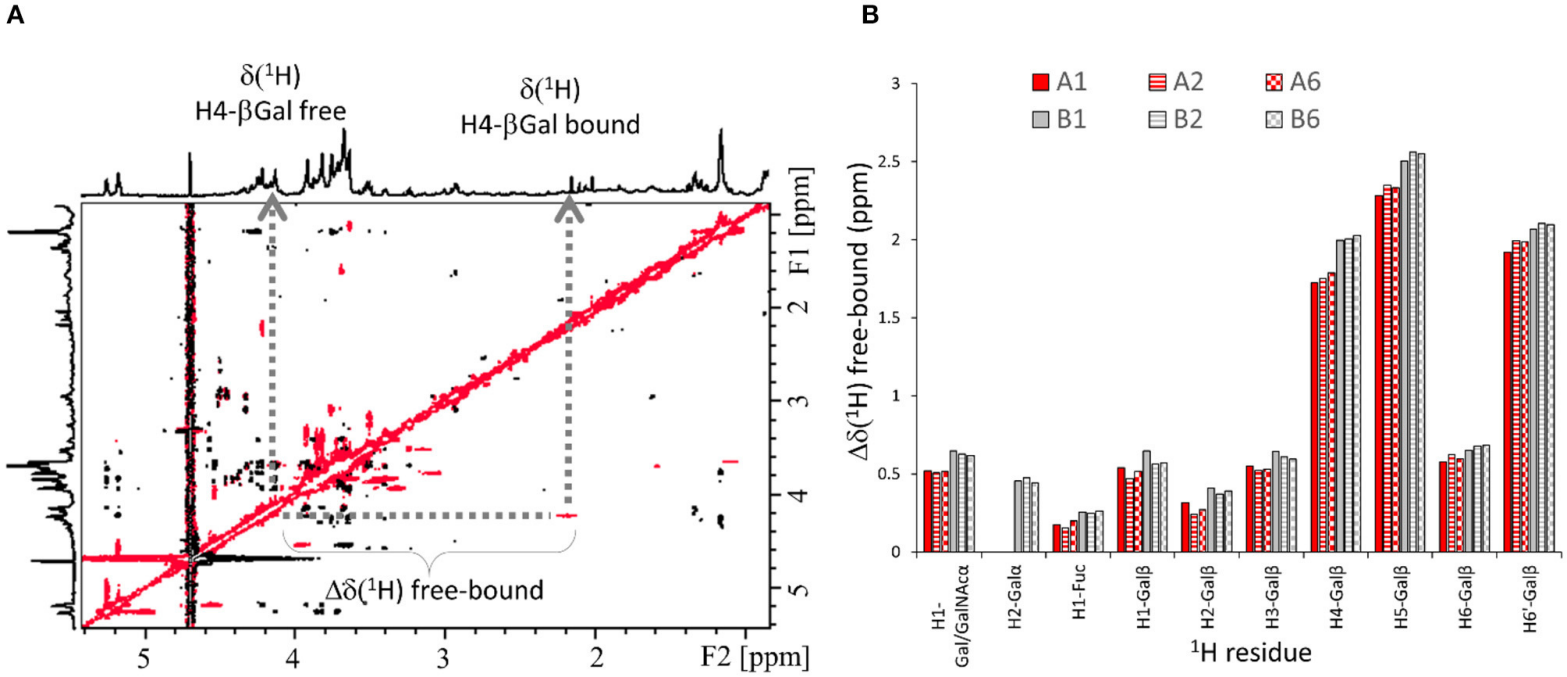
^1^H-NMR-based ligand chemical exchange (EXSY) analysis from the trROESY experiments. **(A)** trROESY spectrum of the B type-6 tetrasaccharide antigen in the presence of Gal-4N (molar ratio 20:1). The cross-peaks in black correspond to those arising from regular nuclear Overhauser effects in the rotating frame, while those in red arise from chemical exchange. Their chemical shifts at the different ^1^H dimensions in the 2D spectrum correspond to those in the free (down-field) and bound (up-field) states. **(B)** Plot for the measured differences in chemical shifts between the free and bound states (Δδ[^1^H] free-bound) for those selected protons of the antigens that experience slow exchange in the ROESY spectrum in the presence of the lectin.

Transverse ROESY (trROESY) spectra were acquired in 1:20 protein/ligand molar ratios (Asensio et al., 1995). The comparison of the observed ROESY cross-peaks with those recorded for the free ligands was very similar, indicating that no significant conformational changes around the glycosidic torsion angles or at the pyranose chairs took place upon binding. Most interestingly, these trROESY spectra evidenced that some ligand protons were in slow chemical exchange between their free and bound states (Figure 3), thus revealing the corresponding chemical shifts in the bound states (Gimeno et al., 2019). Fittingly, those signals experiencing slow exchange in the NMR chemical shift timescale are expected to arise from protons that significantly change their chemical environment upon complex formation and that are highly affected by their proximity to the lectin surface. Remarkably, not all ligand protons showed chemical exchange cross-peaks. The analysis of the chemical shift difference between the free and bound forms [Δδ _(free−bound)_] thus afforded additional epitope mapping information from the ligand perspective. Moreover, the comparison of the behavior observed for the different ligands also permitted the deduction of subtle differences related to their different binding geometries into the galectin binding site. Notably, for all ligands, all the protons of the central βGal residue experienced drastic up-field shifts upon complex formation, with H4, H5, and one of the H6 standing out with more than δ 2 ppm difference between the free and bound forms (Figure 3). This effect is in full agreement with the expected key CH-π stacking interaction (Asensio et al., 2013) between the central βGal residue and the indole side chain of W84 of the lectin. This key βGal/Trp interaction is ubiquitous for all galectins (Dings et al., 2018; Bertuzzi et al., 2020). Nevertheless, the profile is slightly but noticeably different for the A and the B antigens (Figure 3). For the B epitopes, H1 and H2 of the terminal βGal moiety experienced slow exchange, being up-field shifted upon binding. On the contrary, for the A-antigens, only H1 of the analogous βGalNAc residue displayed slow exchange with a reduced chemical shift perturbation, while H2 was not in slow exchange. The chemical shift difference free-bound for H4, H5, and H6′ of the central βGal was also smaller for the A antigens than for the B antigens (Figure 3), suggesting that the stacking interaction is stronger for the latter. Additionally, H1-Fuc (the only proton of the Fuc ring under slow exchange) is more affected in the B antigens [Δδ_(free−bound)_ = 0.2–0.3 ppm] than in the A antigens (<0.2 ppm). These differences strongly suggest that the A and B antigens display a slightly different orientation in the Gal-4N binding site. Additionally, very few protons at the reducing-end residues showed weak chemical exchange effects (see Supplementary Information). Finally, the type-4 oligosaccharides did not show any chemical exchange effect.

#### Chemical Shift Perturbations at Gal-4N

The molecular recognition event was also analyzed from the perspective of the lectin. Thus, a chemical shift perturbation (CSP) analysis of the changes produced in the ^1^H-^15^N HSQC spectra recorded for ^15^N-labeled Gal-4N upon addition of the different ligands was carried out. This analysis required the NH backbone NMR assignment of the lectin signals, following well-established triple resonance 3D NMR methods (see Materials and methods and Supplementary Information). The assignment protocol allowed assigning 80% of the NH backbone resonances. In addition to the ligands shown in Scheme 1, the corresponding non-fucosylated analogs and the H antigens (devoid of the terminal Gal/GalNAc moieties, see Supplementary Information), as well as lactose, were also employed. Thus, a full comparative analysis on the monosaccharide-specific effects on the lectin CSP was achieved. In addition, titration experiments afforded an estimation of the binding affinities, which are provided in Table 1.

**Table 1:** Equilibrium dissociation constants (K_D_) determined for the interactions of the different A and B blood group antigens with Gal-4N by employing HSQC NMR-based titrations and ITC measurements, along with the thermodynamic parameters for the interactions, as determined by ITC.

**Ligand**	**K_**D**_ (μM)**	**K_**D**_ (μM)**	**Δ*G* (kcal mol^**–1**^)**	**Δ*H* (kcal mol^**–1**^)**	**-*T*Δ*S* (kcal mol^**–1**^)**
	**(NMR)**	**(ITC)**	**(ITC)**	**(ITC)**	**(ITC)**
A type-1 (tetra)	440	267	−4.9	−5.9	1.0
A type−2 (tetra)	370	187	−5.1	−6.9	1.8
A type-4 (penta)	1,380	-	-	-	-
A type-6 (tetra)	150	86	−5.6	−8.5	2.9
B type-1 (tetra)	200	109	−5.4	−7.7	2.3
B type-2 (tetra)	190	88	−5.5	−9.3	3.8
B type-4 (penta)	460	-	-	-	-
B type-6 (tetra)	60	51	−5.9	−12.3	6.4

The observed CSP for the interaction with the trisaccharide 2'-fucosyllactose (H type-6) was the same as that for lactose, suggesting that the Fuc residue does not provide direct interactions with the lectin. In contrast, when the non-fucosylated B type-6 antigen was employed, additional CSPs with respect to lactose were observed. These perturbations took place mainly at the S2 strand and at the side chain of W84, evidencing that the terminal αGal unit indeed interacts with this area of Gal-4N (Supplementary Information).

**Figure 4:**
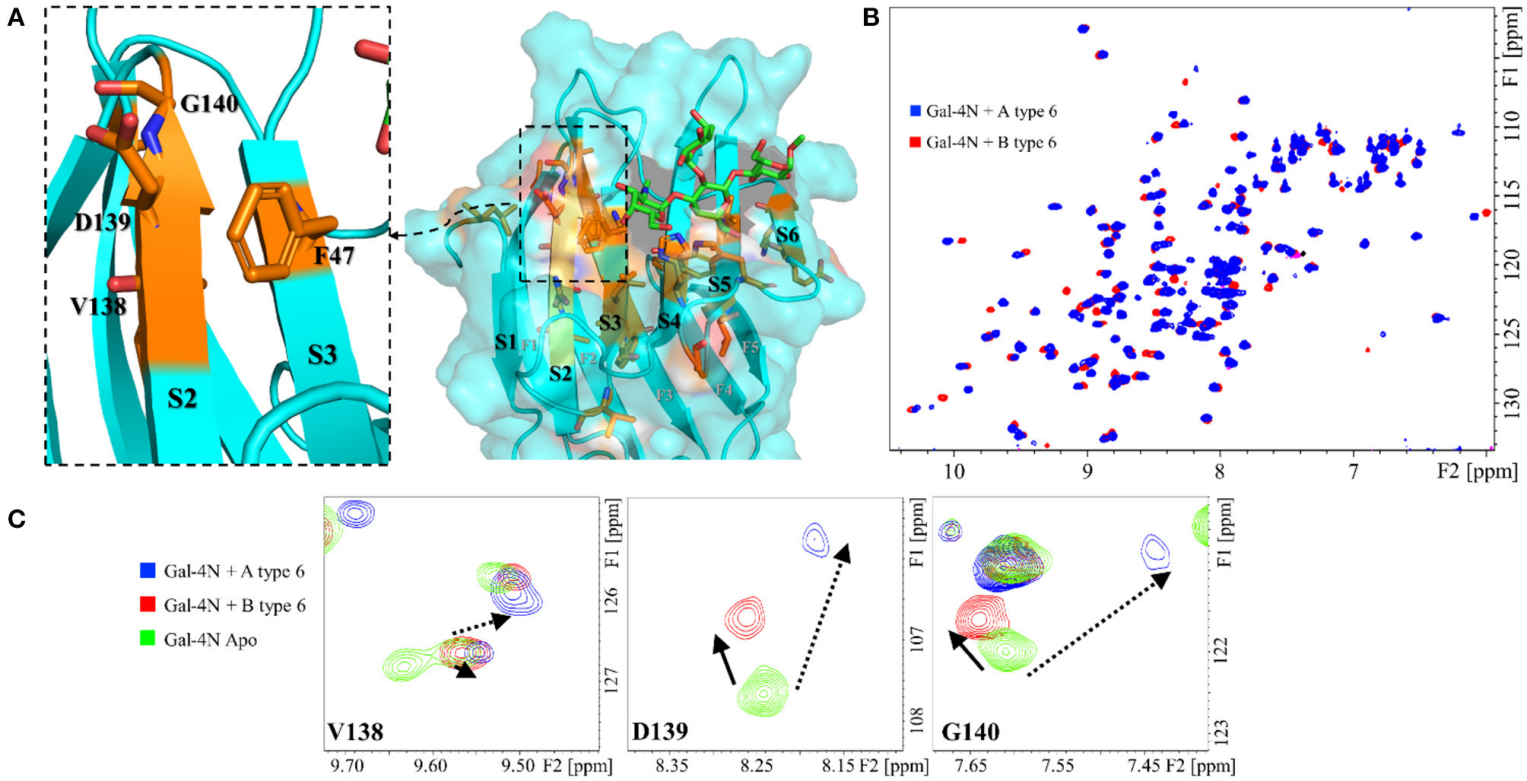
Protein backbone CSP analysis for the interaction of Gal-4N with A and B type-6 tetrasaccharide antigens. **(A)** Molecular model (MD simulation) for the complex of Gal-4N with A type-6 antigen. The residues that significantly differ in their chemical shift perturbation upon binding to group A or B antigens are highlighted in orange, as well as F47 (unknown resonance assignment). Left: zoom at the S2–S3 region showing residues F46, V138, D139, and G140. **(B)** Superimposition of the ^1^H-^15^N HSQC spectra of Gal-4N in the presence of A type-6 (blue) and B type-6 antigens (red). **(C)** Expansion of the ^1^H-^15^N HSQC spectra at residues V138, D139, and G140: in green, Gal-4N apo; in blue, Gal-4N/A type-6 (12 eq.); and in red Gal-4N/B type-6 (10 eq.).

The CSP measured for the A antigens vs. the B antigens showed significant differences for several residues, all located along the S face of Gal-4N (Figure 4). Some of these residues cluster in the S2 strand, such as V138, D139, and G140. These residues are remarkably differently affected by the A and B antigens, both in magnitude and direction: they are more strongly perturbed in the presence of A antigens, moving up-field in the ^1^H dimension, than in the presence of B antigens (Figure 4C). The analysis of the published x-ray crystallographic structures (Bum-Erdene et al., 2015) showed that these amino acid residues are facing F47, located at the contiguous S3 strand. The observed trends suggest that the interaction with the A-antigens moves residue F47 closer to these V138-G140 residues, a motion that does not take place when the B-antigens are bound (Figure 4A).

**Figure 5:**
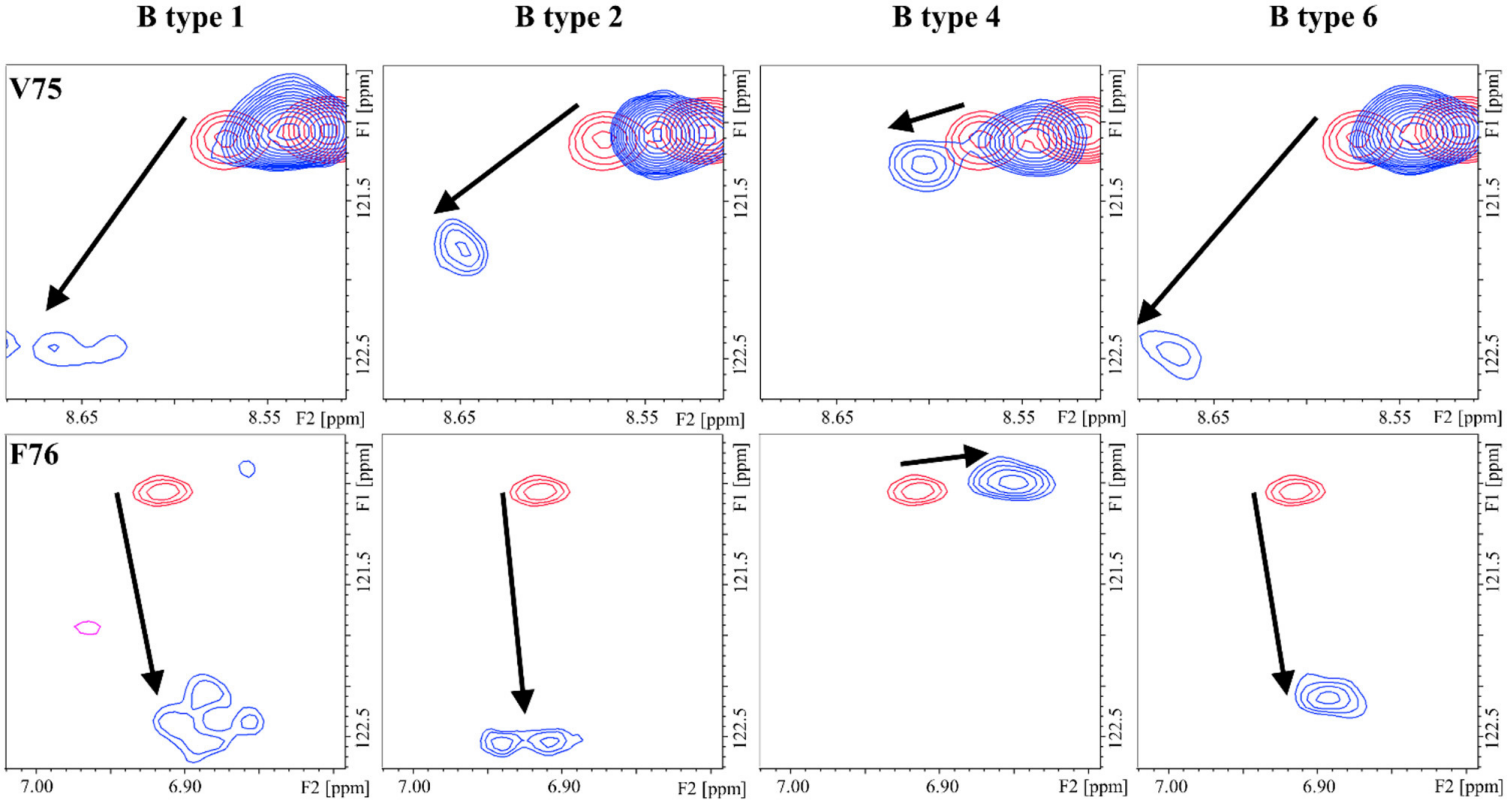
^1^H-^15^N HSQC: chemical shift perturbation of residues V75 and F76 of Gal-4N in the presence of the B antigens types-1, -2, -4, and -6.

The different blood group types also displayed distinct CSP of the lectin cross-peaks. The CSP between types-1 and -2 were basically identical, with only subtle differences at V75 (S5) and R89 (S6) (Supplementary Information), while type-6 oligosaccharides presented different perturbations all along the S5 and S6 strands (Supplementary Information) with the strongest differences involving residues E87, R89, and K90. As for the type-4 antigens, there was a remarkable lack of perturbations at the S5 strand (Figure 5), more pronounced for the B antigen than for the A antigen (Supplementary Information).

In addition to the information yielded by the CSP analysis, titrations provided an estimation of the equilibrium dissociation constants of each ligand (K_D_, Table 1). The NMR-based binding affinities spanned a 20-fold range between the best (B-type 6) and worse (A-type 4) binders, with K_D_ values in the μM to mM range. Lactose, the minimum binding epitope, also exhibited a very low, although measurable, affinity with a K_D_ of 1.6 mM, which is in agreement with previously reported data (Bum-Erdene et al., 2016; Sindrewicz et al., 2019). Interestingly, the tetrasaccharides presented conserved trends in their binding preferences. Thus, for the same peripheral core structure, the B-type antigens were always better binders than the corresponding A-type antigens. With respect to the core structures, type-6 are the best binders, followed by types-1 and -2 that are very similar, irrespective of their terminal residues (whether they are A or B). Finally, the type-4 oligosaccharides were the worse binders, with A type-4 being almost as weak as lactose.

### Isothermal Titration Calorimetry

**Figure 6:**
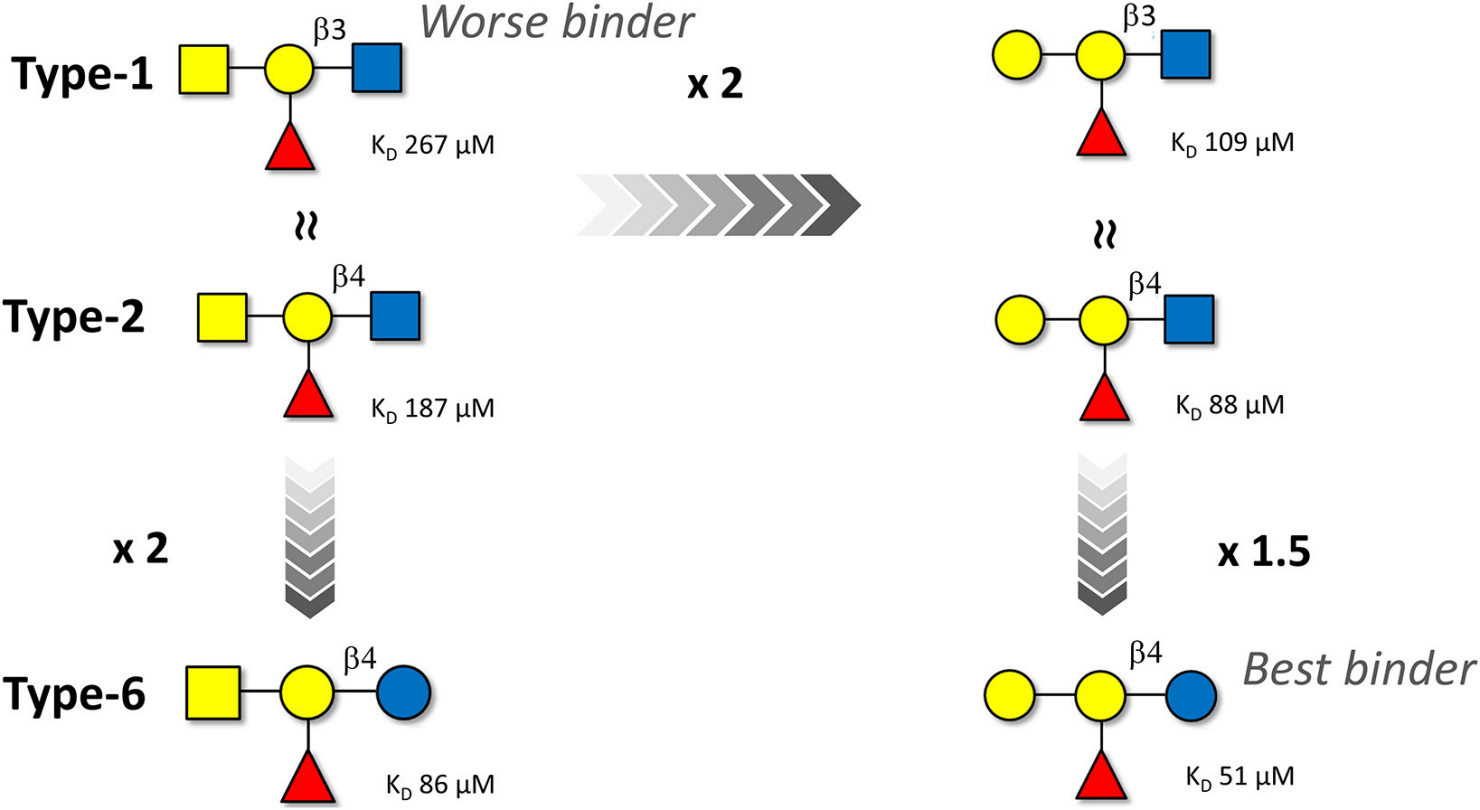
Schematic representation of the affinity trends for the binding of the A and B blood group antigen tetrasaccharides (types-1, -2, and -6) to Gal-4N as deduced by ITC. The NMR-based data follow the same trend although the estimated binding affinities are *ca*. 1.5-fold weaker. The type-4 oligosaccharides are not displayed since their affinities are even weaker than those of the type-1 analogs.

In order to provide alternative and complementary estimations of the binding affinity data, ITC experiments were also carried out for the six HBGA tetrasaccharides (Table 1). The binding with the type-4 pentasaccharides was too weak to obtain reliable ITC data. The thermodynamic profile for all the interactions showed the typical enthalpy-driven binding process (Table 1). The best enthalpy values are always compensated by opposing entropy contributions, especially significant for the B-type 6 oligosaccharide. The significant favorable binding enthalpy (−12.3 kcal/mol) is accompanied by the largest entropy penalty (−6.4 kcal/mol). This binding profile constitutes a prototypical example of enthalpy-entropy compensation with a nearly perfect relationship between Δ*H* and –*T*Δ*S* (see Supporting Information). However, due to the moderate- or low-affinity interactions (all with K_D_ values above 50 μM), the resulting thermodynamic parameters will not be further interpreted (Turnbull and Daranas, 2003). Although the magnitudes of the ITC-derived K_D_ values were on average 1.5-fold lower than those determined by NMR, both techniques yielded conserved trends, reproducing the binding preferences described above; this process is schematized in Figure 6.

The trend is very clear: The B group antigens are better binders than their corresponding A group analogs. Regarding the peripheral disaccharide cores, the type-6 antigens are preferentially recognized, while the type-4 ones display very weak affinity. The type-1 and type-2 antigens display intermediate affinities.

In particular, for β1–4 linked antigens, the substitution of Glc by GlcNAc at the reducing-end is detrimental for the binding event, as evidenced by the comparison between A type-2 and A type-6 and between B type-2 and B type-6. In both cases, a two-fold increased affinity is observed for the type-6 linkages.

In contrast, the presentation of the β1-3GlcNAc vs. β1-4GlcNAc epitopes at the peripheral core (type-1 vs. type-2) basically does not influence the binding affinity for both the A and the B tetrasaccharides.

Finally, the B antigens (with a terminal αGal residue) always bind 2-fold stronger than the A antigens (with a αGalNAc residue), irrespective of the peripheral core disaccharide.

The binding preferences of Gal-4N can be thus be categorized as shown in Figure 6, where the B type-6 is the best binder with a 5-fold difference in affinity. Therefore, Gal-4N prefers αGal moieties at the non-reducing end and Glc residues at the reducing end.

### Three-Dimensional Structural Models for the Binding Complexes

**Figure 7:**
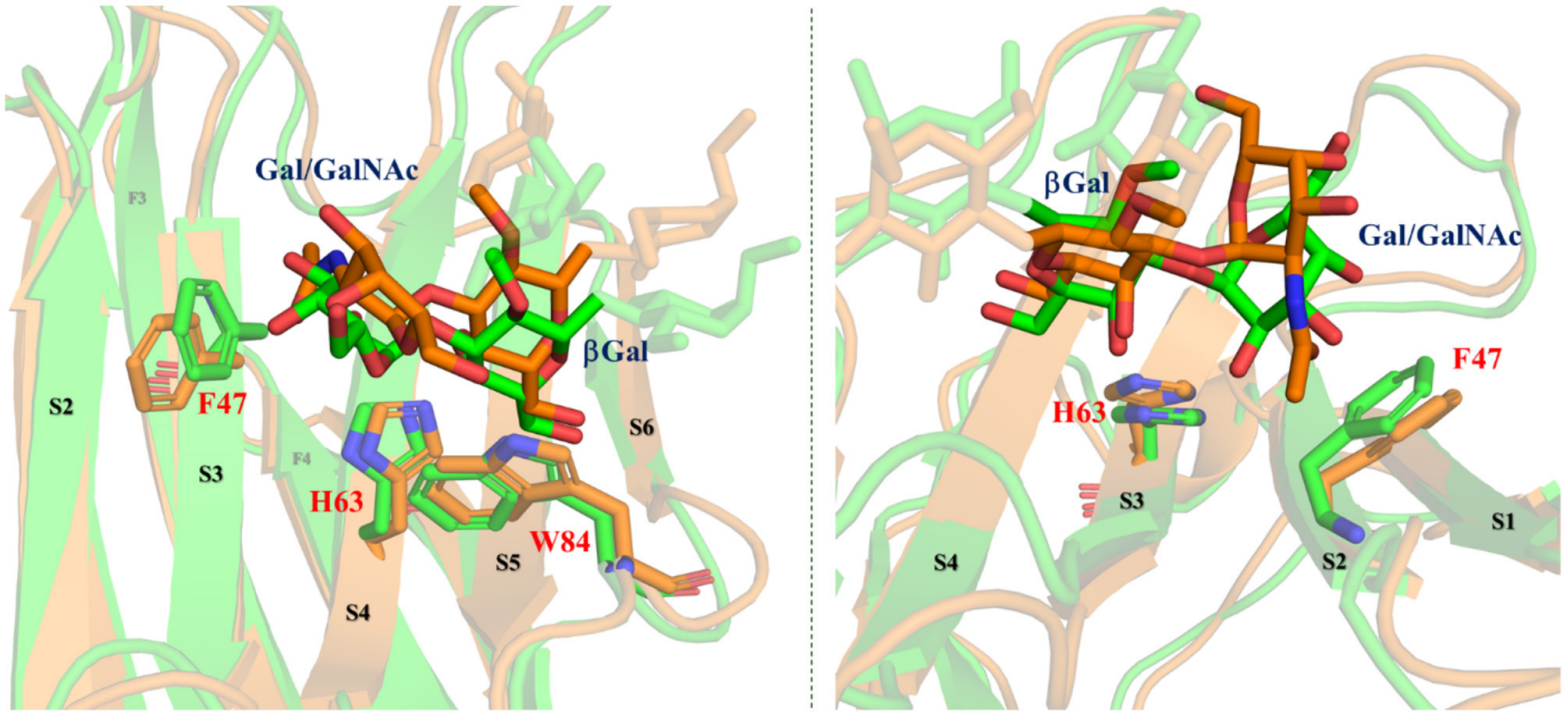
Different perspectives of the superimposition of the 3D models for the complexes Gal-4N/A type-6 (orange) and Gal-4N/B type-6 (green) according to the MD simulations. Reducing-end and Fuc residues are faded. Key residues of the protein are highlighted.

Once the experimental data were available, 3D structural models for the complexes were generated by atom-pair superimposition of the central βGal pyranose of the ligands with the corresponding residue in the x-ray crystallographic structure of the reported complex of Gal-4N bound to lactose (pdb 5DUV) (Bum-Erdene et al., 2015). Then, the generated complexes were submitted to 1 μs molecular dynamics (MD) simulations using the ff14SB and GLYCAM06j-1 force fields as implemented in Amber18. Figure 7 gathers the superimposition of the complexes formed by Gal4-N with the A type-6 and the B type-6 antigens as a summary of the results. Fittingly, the subtle different orientations and presentations of the ligands at the lectin binding site permit the explanation of the observed differences found in the combined ligand-based and receptor-based NMR analysis and the ITC measurements.

As discussed above, the B antigens are better binders (and display better enthalpies) than the A analogs. In fact, according to the MD simulations, only in the A-complex, the bulky Ac group at the terminal GalNAc pushes F47 (in S3) away, moving it toward S2. The lack of this Ac group in the B-complex allows for a better binding. These theoretical predictions are in full agreement with the experimental observations found in the CSP analysis (Figure 3). In fact, for all the A antigens, the αGalNAc residue is further away from the protein surface. For instance, for the group A type-6, H2-αGalNAc is on average at 5.3 Å from the aromatic ring of F47, whereas for the group B type-6, H2-αGal is only 3.5 Å away from the aromatic ring of F47. This fact perfectly matches with the chemical exchange observations in the trROESY experiments in which this proton is up-field shifted only for the B antigens. The ring current effect of the F47 aromatic ring affects H2-αGal due to its close proximity and orientation (Figure 7). Regarding the central βGal moiety, given the diverse presentation and fitting of the terminal residues, this βGal residue in the A and B antigens is also differently positioned with respect to the amino acids that comprise the binding site (Figure 7). In fact, in the A antigens, H4-βGal is 0.5 Å further away from the imidazole ring of H63 than in the B antigens. Additionally, H5-βGal is also 0.4 Å further away from the indole ring of W84 in the A antigens than in the B antigens. These different geometries also correlate with the observed stronger chemical shift perturbations of these protons in the B antigens than in A antigens. These geometric differences also impact both the strength of the βGal/W84 CH-π interaction as well as the OH4-βGal/H63 hydrogen bond, which are key interactions in the molecular recognition of βGal moieties by galectins.

**Figure 8:**
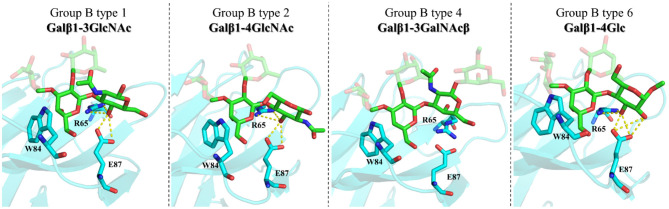
Molecular models of the complexes between Gal-4N and group B types-1, -2, -4, and -6 antigens according to MD simulations. Key residues are highlighted.

With respect to the core-disaccharides, the MD simulations show that, in the β1–4 linked epitopes (types-2 and -6), positions 2 and 3 of the reducing-end pyranose ring are facing the lectin, while in β1-3 linked analogs (types-1 and -4), these positions are in the opposite direction, exposed to the bulk solvent (Figure 8). These MD-based orientations are also in full agreement with the STD data discussed above. For the β1-4 linked epitopes, the MD simulations also predict hydrogen-bonding interactions between OH3 of the reducing end sugar (Glc/GlcNAc) and the polar side chains of R65 and E87. Also, only in the type-6 antigens, an additional hydrogen bond is established between OH2-Glc and the carboxylate group of E87. This additional stabilizing interaction could provide the impetus for the additional affinity gain observed in the type-6 vs. the type-2 cores. In type-1 antigens, which are β1-3 linked, R65 and E87 establish hydrogen bonding with OH4-GlcNAc. Thus, despite the different orientations of the reducing-end pyranose with respect to β1-4 linked antigens, type-1 and type-2 would be balanced in terms of establishing interactions, which is in agreement with their similar affinities. Finally, for type-4 antigens, the axial disposition of the OH4-GalNAcβ precludes the interactions with R65 and E87, which is in agreement with their reduced affinity.

## Conclusions

Herein, the binding preferences of the N-terminal of Gal-4 toward A and B blood group antigens have been examined at the molecular level. Different antigenic presentations in terms of peripheral core structures have been compared, as well as the A vs. B antigen preference. The ITC experiments permitted the classification of the ligands in terms of binding affinity, while different NMR strategies in combination with modeling protocols provided the structural rationale for the observed binding preferences. All the ligands bound Gal-4N with similar affinities, with K_D_ values in the range of 10^−5^−10^−4^ M for the tetrasaccharides and a 7-fold difference between the best and worse ligands, while the pentasaccharides bound weaker by one order of magnitude (K_D_ values in the range of 10^−4^−10^−3^ M). Conserved binding trends were observed. For the same peripheral core structure, the B antigen was always a better binder than the A antigen, indicating that the NAc group on the terminal αGal residue is detrimental for the binding. A similar effect was found for β1-4 linked antigens at the peripheral core, for which the presence of the NAc in type-2 antigens (β1-4GlcNAc) was again detrimental for the binding with respect to type-6 antigens (β1-4Glc). The type-1 and type-2 antigens, despite the different orientation of their reducing-end residues with respect to the protein, displayed similar affinities.

The experimental NMR observations, both from the ligand and lectin perspectives, could be nicely reconciled with the molecular models for the complexes obtained through MD simulations, where the slightly different chemical modifications among the ligands provoked subtle structural differences in the complexes, with diverse impacts in the binding affinities. These data might help in understanding the binding preferences of Gal-4 toward the histo blood group antigens, especially with respect to their different presentations in distinct biological contexts (e.g., tissues) or their diverse origins (self vs. pathogenic). In addition, this study provides detailed atomic-level information and dynamic features about Gal-4N binding characteristics, which are relevant in the context of rational ligand design.

## Materials and Methods

### Ligands

Lactose was purchased from Sigma (St. Louis, MO, United States). Glycans H type-6, B type-6 trisaccharide, A types-1, -2, and -6 tetrasaccharides, A type-4 pentasaccharide, B types-1, -2, and -6 tetrasaccharides, and B type-4 pentasaccharide were purchased from Elicityl (Crolles, France) (references GLY031-3, GLY074-2, GLY035-1, GLY035-2, GLY035-3, GLY128, GLY038-1, GLY038-2, GLY038-3, GLY129).

### Protein Expression and Purification of Gal-4N

The DNA fragment coding for the N-terminal CRD of *h*Galectin-4 (amino acids 1–150, including an additional C-terminus His-Tag) was inserted into the pET29b expression vector, amplified in *E. coli* DH5α cells, and afterward transformed into *E. coli* BL21 cells. A single colony was inoculated into 200 ml Luria Broth (LB) medium containing 50 μg ml^−1^ kanamycin and was cultured at 37°C overnight. A precise quantity of the cultured colony was then added to 2 L of fresh LB containing kanamycin to achieve an initial optical density at 600 nm (OD600) of 0.1. The cells were grown at 37°C until the OD600 reached 0.6–0.8; subsequently, protein expression was induced by addition of 1 mM of isopropyl-1-thio-β-D-galactopyranoside (IPTG), and the culture was allowed to grow overnight at 20°C. For the ^15^N-labeled and ^13^C,^15^N-labeled samples, a single colony was inoculated into 5 ml of LB and was cultured for 6 h at 37°C. The small preculture was centrifuged and resuspended in 1 ml of M9 medium, transferred to 200 ml of M9 medium, and then incubated overnight at 37°C. A precise quantity of the culture was then added to 2 L of fresh M9 medium containing 1 g/L ^15^NH_4_Cl for the ^15^N-labeled samples, or 1 g/L ^15^NH_4_Cl and 20% w/v U–^13^C-glucose for the ^13^C,^15^N-labeled samples. Unlabeled amino acids (L-Arginine, L-Phenylalanine, L-Valine, or L-Lysine) were added in the quantity of 1 g/L to the M9 medium containing 1 g/L ^15^NH_4_Cl to selectively not label concrete amino acids of the protein. The culture was harvested by centrifugation at 5,000 rpm and the final pellet was resuspended in lysis buffer (50 mM sodium phosphate, pH 8, 300 mM NaCl, 1 mM phenylmethylsulfonyl fluoride) and sonicated at 4°C. The crude extract was clarified by centrifugation at 35,000 rpm for 1 h at 4°C. The soluble fraction was purified by Ni-NTA (Sigma, St. Louis, MO, United States) affinity chromatography and further purified by size exclusion chromatography in a HiLoad 26/600 Superdex 75 column (Sigma, St. Louis, MO, United States). The C-terminal His-Tag was removed by overnight incubation at 4°C with thrombin (10 units of thrombin per mg of protein). Afterward, the protein was loaded onto a 5 ml Ni-NTA column, and the desired fragment was collected in the wash and loaded in a HiLoad 26/600 Superdex 75 column. Gal-4N purity was checked by 4–12% SDS-PAGE (Thermo Fischer, Waltham, MA, United States) and by LC-MS.

### NMR Experiments. General Information

The total volume for the NMR samples was 500/μl. The lectin was in phosphate-buffered saline (50 mM sodium phosphate, 150 mM NaCl, pH 7.4), either in D_2_O or 90:10 H_2_O:D_2_O depending on the NMR experiment. The pH was adjusted with the required amount of NaOH and HCl or NaOD and DCl.

### Backbone Resonance Assignment

The backbone resonance assignment of the N-terminal CRD of *h*Galectin-4 was performed at 25°C on an 800 MHz Bruker spectrometer equipped with a cryoprobe (Bruker, Billerica, MA, United States). 3D HNCO, HN(CA)CO, HN(CO)CACB, and HNCACB experiments were performed and assigned for the free Gal-4N containing the His-Tag and for Gal-4N without the His-Tag in the presence of 200 equivalents of lactose. Additionally, HN(CO)CA and HNCA experiments were recorded for the free protein containing the His-Tag. The entire analysis provided the unambiguous identification of 80% of the expected NH signals for Gal-4N. The spectra were processed with Bruker TopSpin 3.5.2 (Bruker, Billerica, MA, United States) and analyzed *via* CARA NMR 1.9.1.4.

### Saturation Transfer Difference NMR

All the STD NMR experiments were performed using an 800 MHz Bruker spectrometer with a cryoprobe (Bruker, Billerica, MA, United States). The samples were prepared in the corresponding deuterated buffer. The temperature was 288 K for every experiment. An amount of 50 μM of the lectin with 50 equivalents of the ligand was employed for every experiment except for lactose, for which a 1:70 ratio was used. The STD spectra were acquired with 1,024 scans, 2 s of saturation time using a train of 50 ms Gaussian-shaped pulses, and 3 s of relaxation delay. The spin-lock filter applied to remove the signals of the lectin was set at 40 ms. The on-resonance frequency was set for the aromatic region at 6.58 ppm, while the off-resonance frequency was set at 100 ppm.

### ^1^H-^15^N-HSQC-Based Titrations

experiments were acquired using an 800 MHz Bruker spectrometer with a cryoprobe (Bruker, Billerica, MA, United States). The samples were prepared using 50 μM of the ^15^N-labeled lectin in the corresponding buffer in a 90:10 H_2_O:D_2_O ratio. The experiments were performed at 298 K. Six to nine points were recorded for each ligand and CSP and K_D_ were calculated using CcpNmr Analysis 2.4.2.

### Transverse ROESY Spectra

The NMR experiments were acquired using an 800 MHz Bruker spectrometer with a cryoprobe (Bruker, Billerica, MA, United States). The ROESY spectra for the glycans were acquired in the presence of 50 μM Gal-4N with a 1:20 protein:ligand ratio in the corresponding deuterated PBS buffer at 298 K.

### Isothermal Titration Calorimetry

Isothermal titration calorimetry experiments were performed using the MicroCal PEAQ-ITC calorimeter (Malvern Panalytical, Malvern, United Kingdom). Samples containing 100–200 μM of Gal-4N in PBS (50 mM sodium phosphate, pH 7.4, 300 mM NaCl) were titrated with stocks of 3–10 mM in PBS of glycans 4, 5, 7, 8, 9, and 11. During the automated experiment, small aliquots (2–3 μL) of the sugar stocks were added to the cell containing the lectin. Curve fitting to a single binding site model was performed with the MicroCal Origin 7 software.

### Molecular Dynamics Simulations

Initial complexes were built by pair-fitting the atomic coordinates of the central βGal moiety of each studied ligand to those of the βGal unit of the bound lactose in the ctystallographic structure (pdb 5DUV). The glycan structures were built in the GLYCAM-Web software. The MD simulations were run using Amber18 with the ff14SB force field for the protein and GLYCAM06j-1 for the carbohydrates. The complexes were prepared in explicit water (TIP3PBOX) and minimized in two steps before starting the simulations. The MD simulations of 1 μs were analyzed using cpptraj.

## Data Availability Statement

The raw data supporting the conclusions of this article will be made available by the authors, without undue reservation.

## Author Contributions

JQ performed all the experiments and wrote the initial draft. SD designed the construct, performed the lectin expression, and purification. RN-F performed the MD simulations, under the supervision of GJ-O, writing the corresponding part. FJC supervised the 3D experiments performed at Madrid and discussed the outcomes. JJ-B and AA designed and supervised the research, discussed the results with all the team, and wrote the final manuscript. All authors contributed to the article and approved the submitted version.

## Conflict of Interest

The authors declare that the research was conducted in the absence of any commercial or financial relationships that could be construed as a potential conflict of interest.
