# Supplementary material for: Galectin-4 N-Terminal Domain: Binding Preferences Toward A and B Antigens With Different Peripheral Core Presentations
Source: Front Chem. 2021 Apr 21;9:664097. doi: 10.3389/fchem.2021.664097 (PMC8097242; doi:10.3389/fchem.2021.664097)
Supplement: Supplementary file 1 [file Data_Sheet_1.docx]

Supplementary Material

**NMR experiments**

**General information**

The total volume for the NMR samples was 500 μL. The pH of the buffer was adjusted with the required amount of NaOH and HCl or NaOD and DCl.

**Saturation Transfer Difference (STD-NMR)**

All the STD experiments were acquired using a 800 MHz Bruker spectrometer with a cryoprobe. The samples were prepared in deuterated phosphate-buffered saline (50 mM sodium phosphate, 150 mM NaCl, pH 7.4). The temperature of the acquisition was 288 K for every experiment. 50 µM of the lectin with 50 equivalents of the ligand was employed for every experiment. STD spectra were acquired with 1024 scans, 2 s of saturation time using a train of 50 ms Gaussian‐shaped pulses and 3 s of relaxation delay. The spin-lock filter applied to remove the signals of the lectin was set at 40 ms. The on-resonance frequency was set for the aromatic region at 6.58 ppm, while the off-resonance frequency was set at 100 ppm.


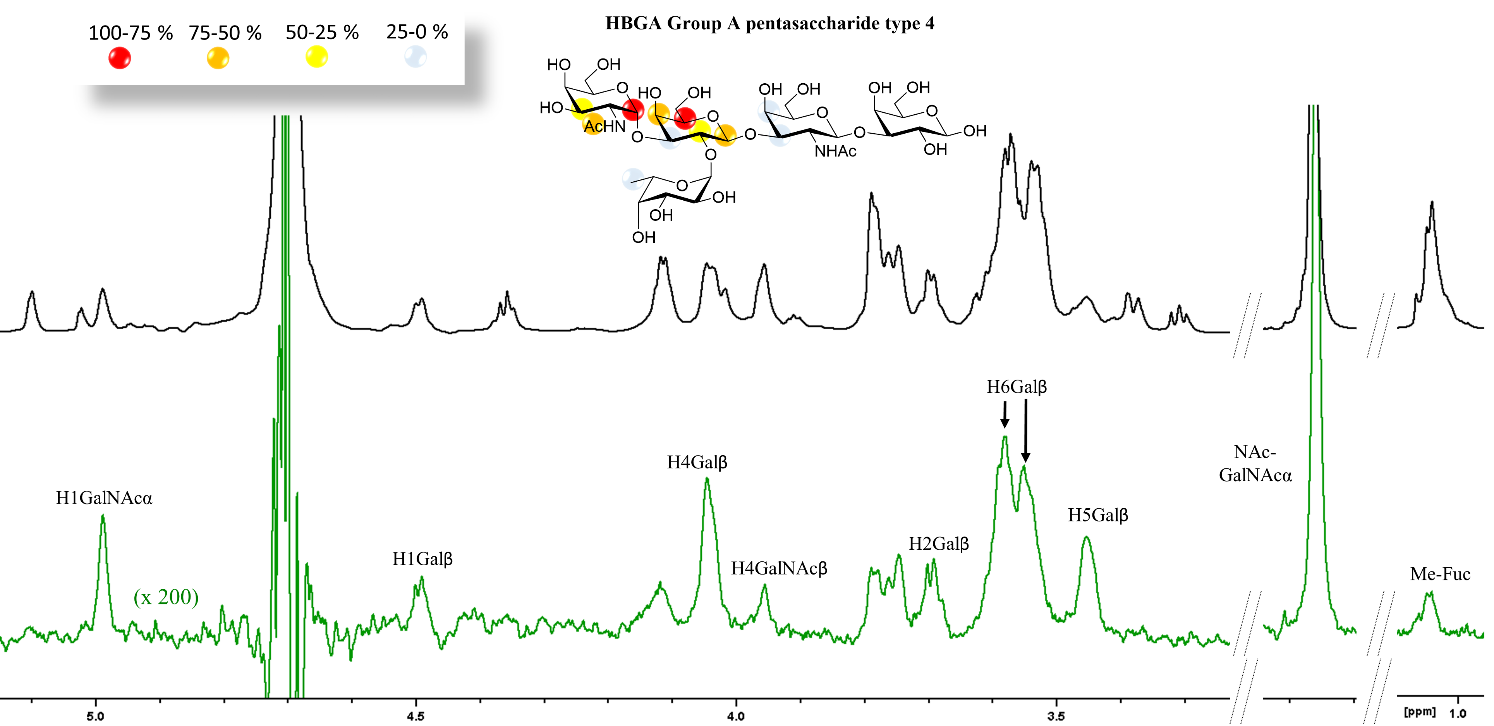


**Supplementary Figure S1.** ^1^H STD-NMR for a sample of group A type-4 pentasaccharide and Gal-4N (50:1 molar ratio). Top: the reference spectrum (black, off-resonance). Bottom: the STD-NMR spectrum (green). The ^1^H-NMR signals showing STD effect are annotated. The epitope mapping (relative STD) is shown in the ligand structure.


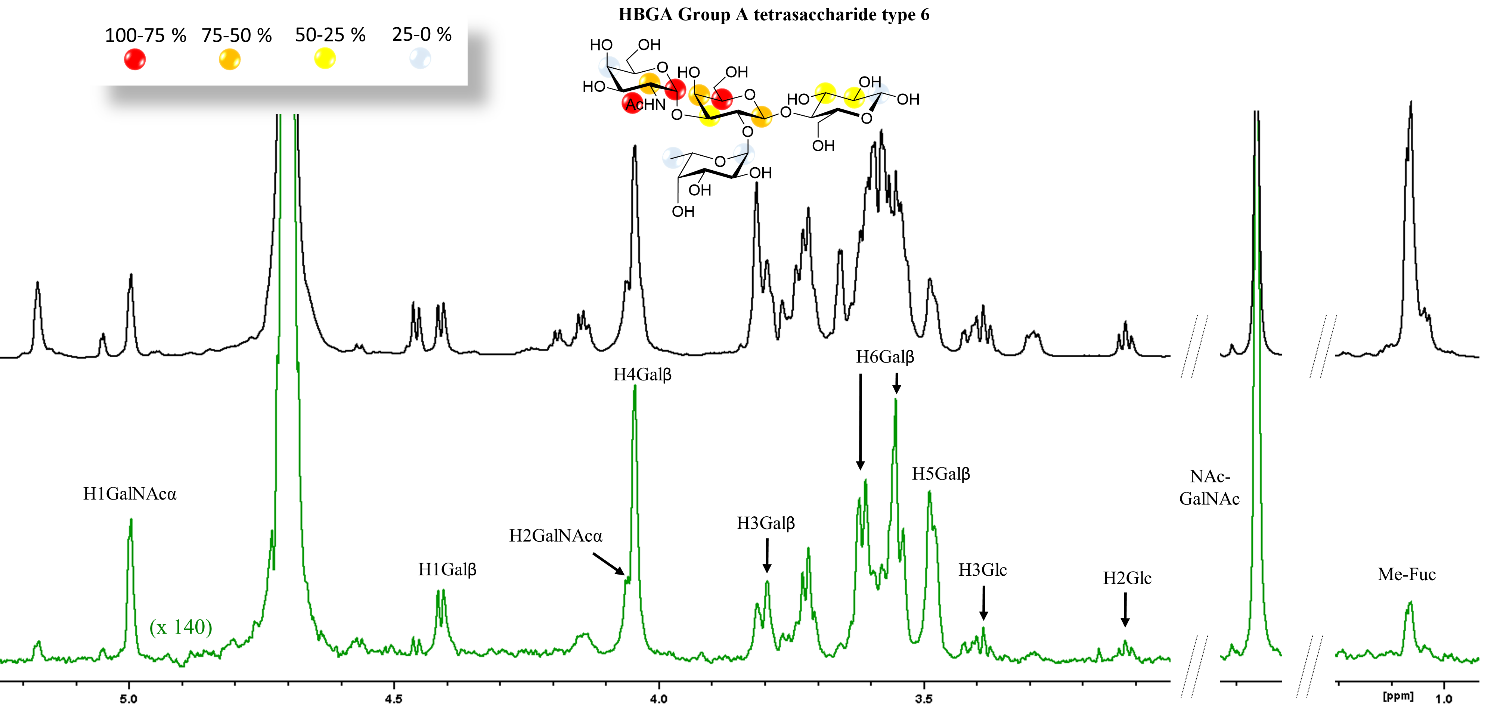


**Supplementary Figure S2.** ^1^H STD-NMR for a sample of group A type-6 tetrasaccharide and Gal-4N (50:1 molar ratio). Top: the reference spectrum (black, off-resonance). Bottom: the STD-NMR spectrum (green). The ^1^H-NMR signals showing STD effect are annotated. The epitope mapping (relative STD) is shown in the ligand structure.


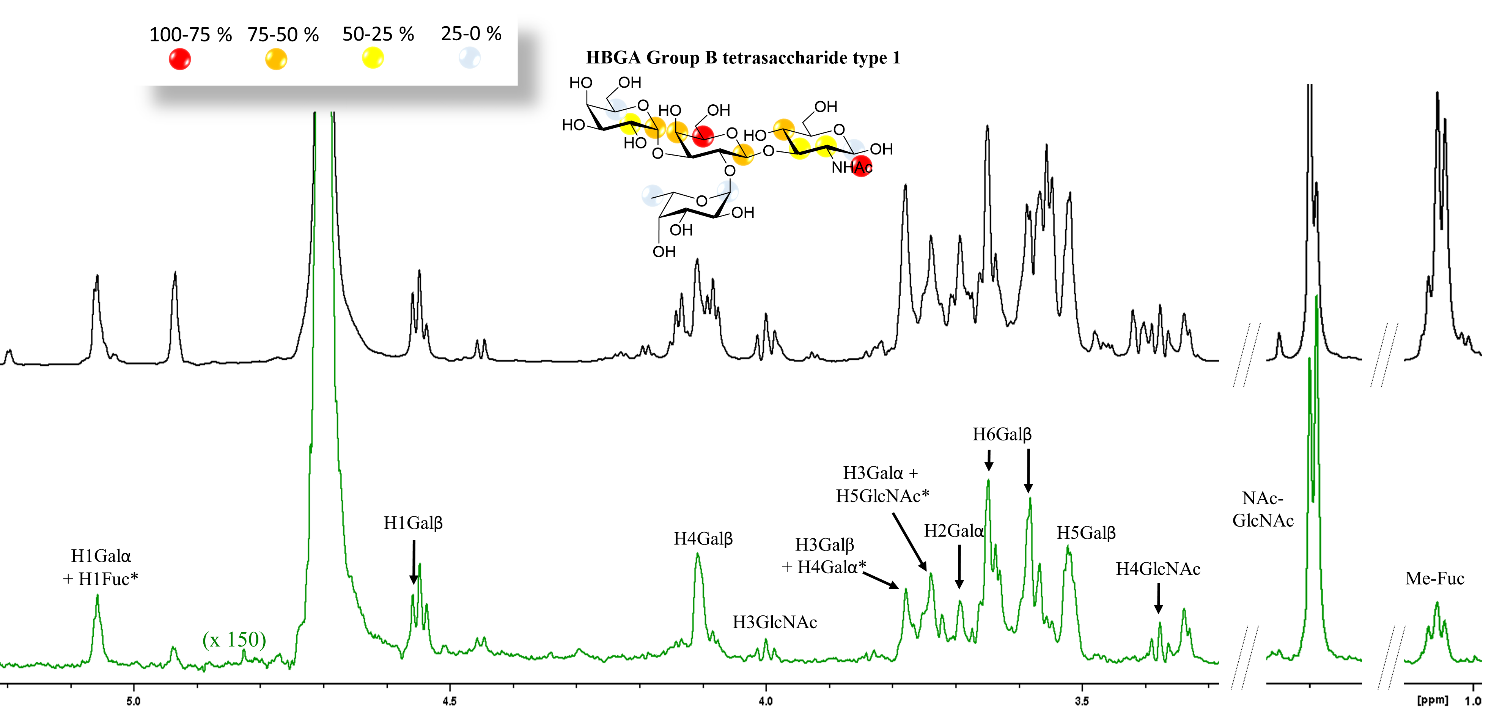


**Supplementary Figure S3.** ^1^H STD-NMR for a sample of group B type-1 tetrasaccharide and Gal-4N (50:1 molar ratio). Top: the reference spectrum (black, off-resonance). Bottom: the STD-NMR spectrum (green). The ^1^H-NMR signals showing STD effect are annotated. The epitope mapping (relative STD) is shown in the ligand structure. * overlapping resonances.


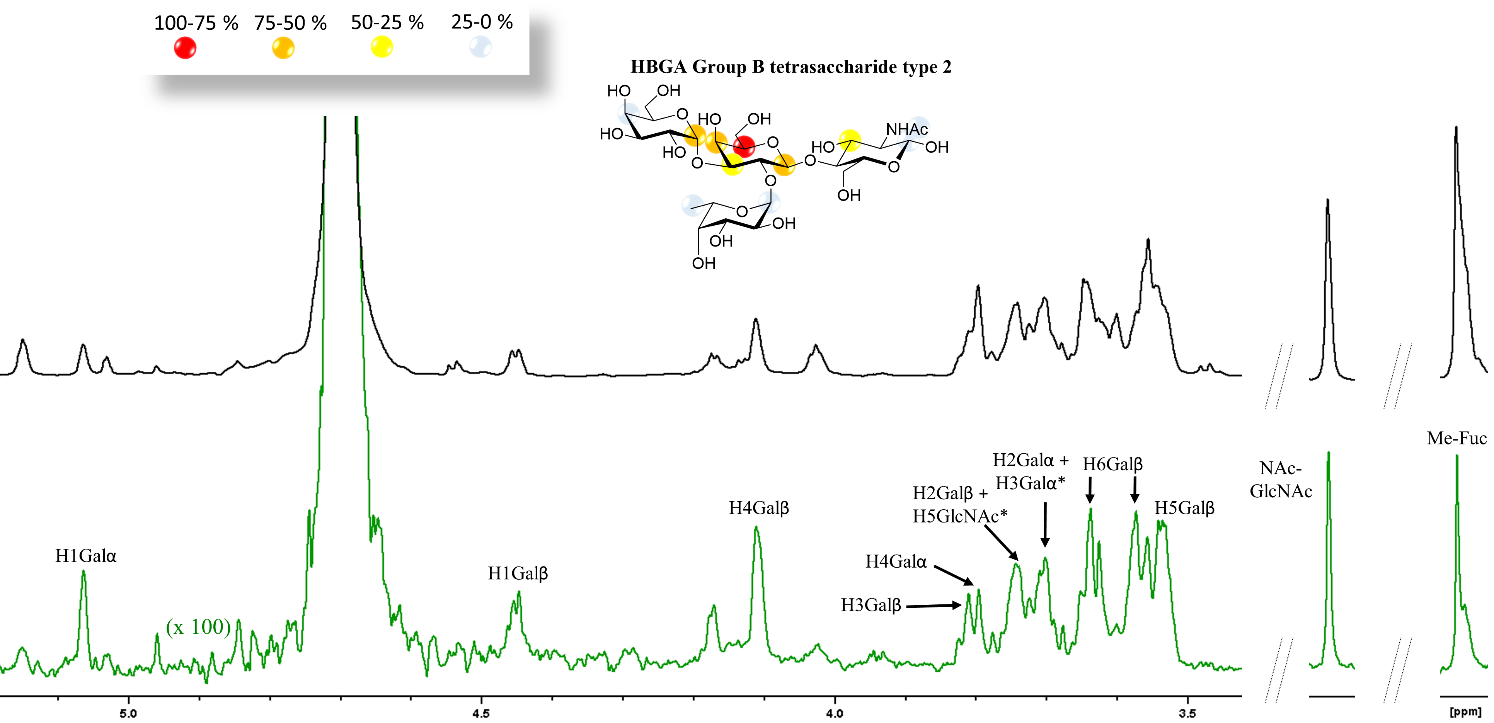


**Supplementary Figure S4.** ^1^H STD-NMR for a sample of B type-2 tetrasaccharide and Gal-4N (50:1 molar ratio). Top: the reference spectrum (black, off-resonance). Bottom: the STD-NMR spectrum (green). The ^1^H-NMR signals showing STD effect are annotated. The epitope mapping (relative STD) is shown in the ligand structure. * overlapping resonances.


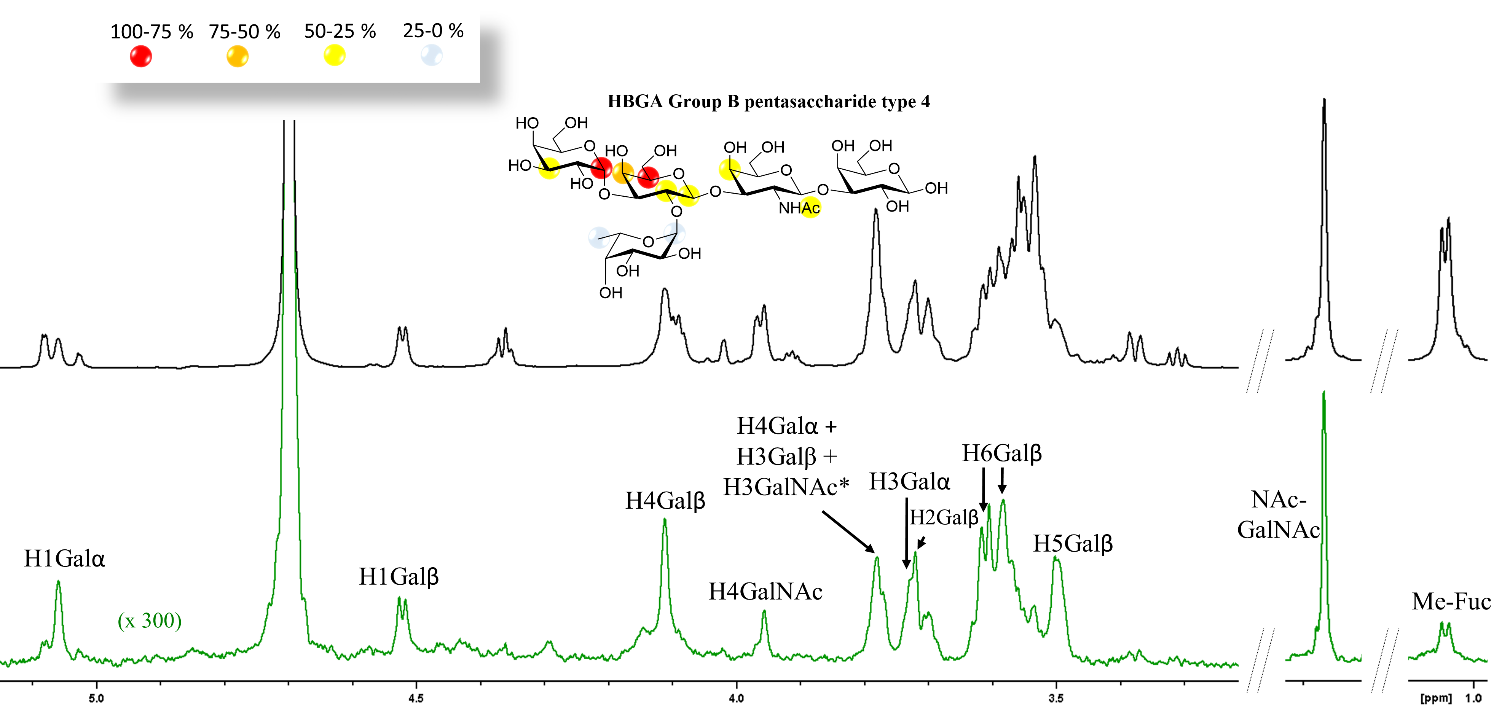


**Supplementary Figure S5.** ^1^H STD-NMR for a sample of group B type-4 pentasaccharide and Gal-4N (50:1 molar ratio). Top: the reference spectrum (black, off-resonance). Bottom: the STD-NMR spectrum (green). The ^1^H-NMR signals showing STD effect are annotated. The epitope mapping (relative STD) is shown in the ligand structure. * overlapping resonances.


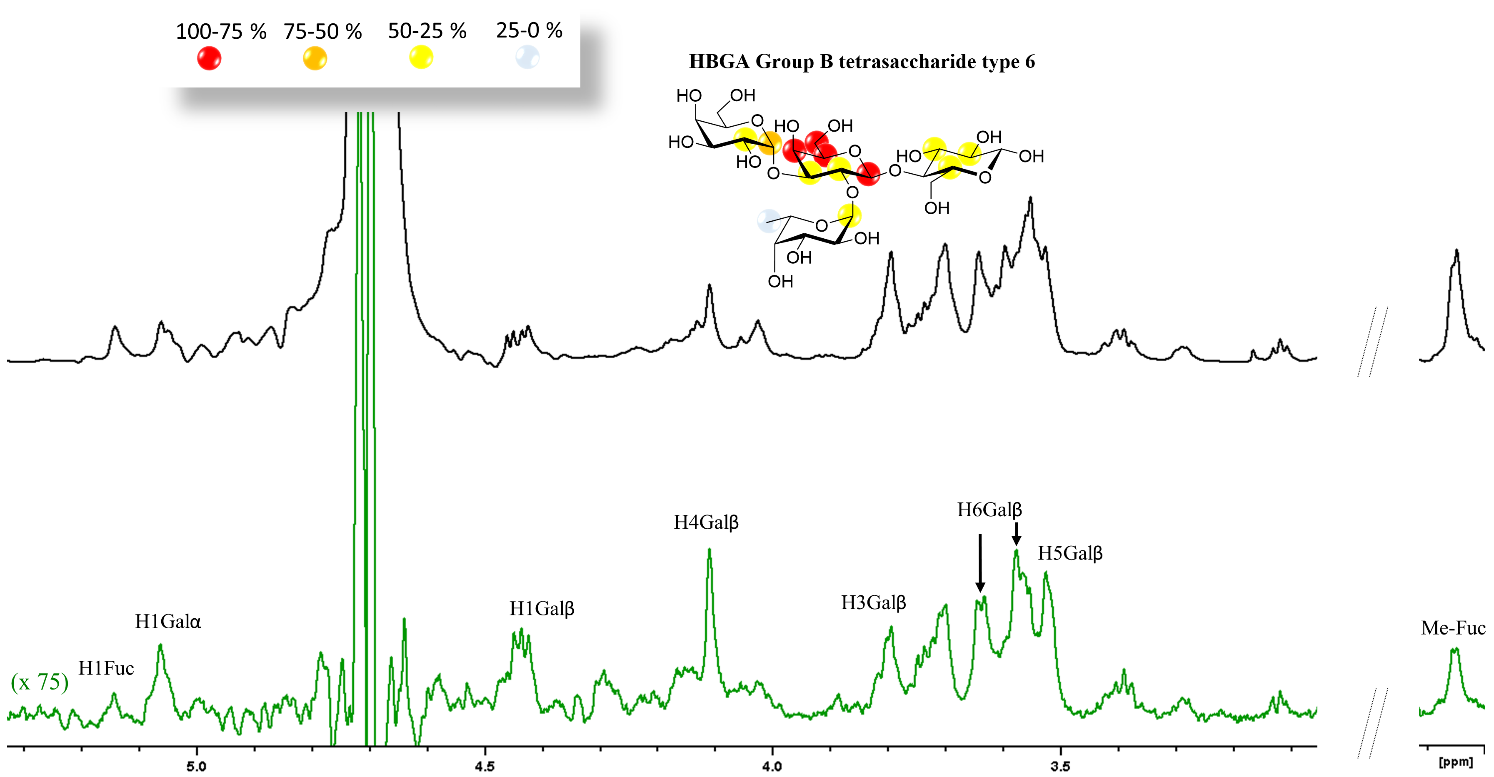


**Supplementary Figure S6.** ^1^H STD-NMR for a sample of group B type-6 tetrasaccharide and Gal-4N (50:1 molar ratio). Top: the reference spectrum (black, off-resonance). Bottom: the STD-NMR spectrum (green). The ^1^H-NMR signals showing STD effect are annotated. The epitope mapping (relative STD) is shown in the ligand structure.

| **Ligand** | **^1^H-residue** | **STD-AF** | **STD%** |
| --- | --- | --- | --- |
| **A type 4** | H1GalNAc | 0.0145 | 95% |
|  | H3GalNAc | 0.0043 | 28% |
|  | NHAc GalNAc | 0.0104 | 68% |
|  | H1Galβ | 0.0092 | 61% |
|  | H2Galβ | 0.0054 | 36% |
|  | H3Galβ | 0.0032 | 21% |
|  | H4Galβ | 0.0108 | 71% |
|  | H5Galβ | 0.0152 | 100% |
|  | H3GalNAcb | 0.0023 | 15% |
|  | H4GalNAcb | 0.0036 | 24% |
|  | MetFuc | 0.0012 | 18% |
| **A type 6** | H1GalNAc | 0.0129 | 82% |
|  | H2GalNAc | 0.0081 | 51% |
|  | H4GalNAc | 0.0025 | 16% |
|  | NHAcGalNAc | 0.0158 | 100% |
|  | H1Galβ | 0.0091 | 58% |
|  | H3Galβ | 0.0061 | 39% |
|  | H4Galβ | 0.0095 | 60% |
|  | H5Galβ | 0.0158 | 100% |
|  | H1Glca | 0.0033 | 21% |
|  | H1Glcb | 0.0028 | 18% |
|  | H2Glcb | 0.0048 | 30% |
|  | H2Glca | 0.0054 | 34% |
|  | H3Glcb | 0.0050 | 32% |
|  | H1 Fuc | 0.0020 | 13% |
|  | MetFuc | 0.0016 | 10% |
| **B type 1** | H1Galα | 0.0053 | 56% |
|  | H2Galα | 0.0033 | 35% |
|  | H5Galα | 0.0022 | 23% |
|  | H1Galβ | 0.0066 | 70% |
|  | H4Galβ | 0.0070 | 74% |
|  | H5Galβ | 0.0094 | 100% |
|  | H1GlcNAc | 0.0015 | 16% |
|  | H2GlcNAc | 0.0027 | 29% |
|  | H3GlcNAc | 0.0036 | 38% |
|  | H4GlcNAc | 0.0050 | 53% |
|  | NHAcGlcNAc | 0.0133 | 141% |
|  | NHAcGlcNAc | 0.0055 | 59% |
|  | Average NHAc | 0.0094 | 100% |
|  | H1 Fuc | 0.0000 | 0% |
|  | MetFuc | 0.0013 | 14% |
| **B type 2** | H1Galα | 0.0108 | 73% |
|  | H4Galα | 0.0027 | 18% |
|  | H1Galβ | 0.0088 | 59% |
|  | H3Galβ | 0.0054 | 36% |
|  | H4Galβ | 0.0081 | 55% |
|  | H5Galβ | 0.0148 | 100% |
|  | H1GlcNAc | 0.0035 | 24% |
|  | H3GlcNAc | 0.0054 | 36% |
|  | NHAc GlcNAc | 0.0035 | 9% |
|  | H1 Fuc | 0.0024 | 16% |
|  | MetFuc | 0.0023 | 16% |
| **B type 4** | H1Galα | 0.0094 | 100% |
|  | H3Galα | 0.0041 | 44% |
|  | H1Galβ | 0.0046 | 49% |
|  | H2Galβ | 0.0044 | 47% |
|  | H4Galβ | 0.0064 | 68% |
|  | H5Galβ | 0.0081 | 86% |
|  | H4GalNAc | 0.0028 | 30% |
|  | NHAc | 0.0034 | 36% |
|  | H1 Fuc | 0.0020 | 21% |
|  | MetFuc | 0.0009 | 10% |
| **B type 6** | H1Galα | 0.0276 | 74% |
|  | H2Galα | 0.0142 | 38% |
|  | H1Galβ | 0.0298 | 80% |
|  | H2Galβ | 0.0129 | 34% |
|  | H3Galβ | 0.0108 | 29% |
|  | H4Galβ | 0.0307 | 82% |
|  | H5Galβ | 0.0374 | 100% |
|  | H2Glcβ | 0.0135 | 36% |
|  | H2Glcα | 0.0146 | 39% |
|  | H3Glcβ | 0.0172 | 46% |
|  | H5Glcβ | 0.0163 | 44% |
|  | H1 Fuc | 0.0105 | 28% |
|  | MetFuc | 0.0074 | 20% |

**Supplementary Table S1.** Relative STD-AF (Amplification factor) and STD percentage of each non overlapping proton of each ligand.

**trROESY NMR**. The experiments were acquired using a 800 MHz Bruker spectrometer with a cryoprobe. ROESY spectra for HBGA tetrasaccharides and pentasaccharides were acquired in the presence of 50 µM Gal-4N with a 20:1 molar ratio in deuterated PBS (50 mM sodium phosphate, 150 mM NaCl, pH 7.4) at 298 K.


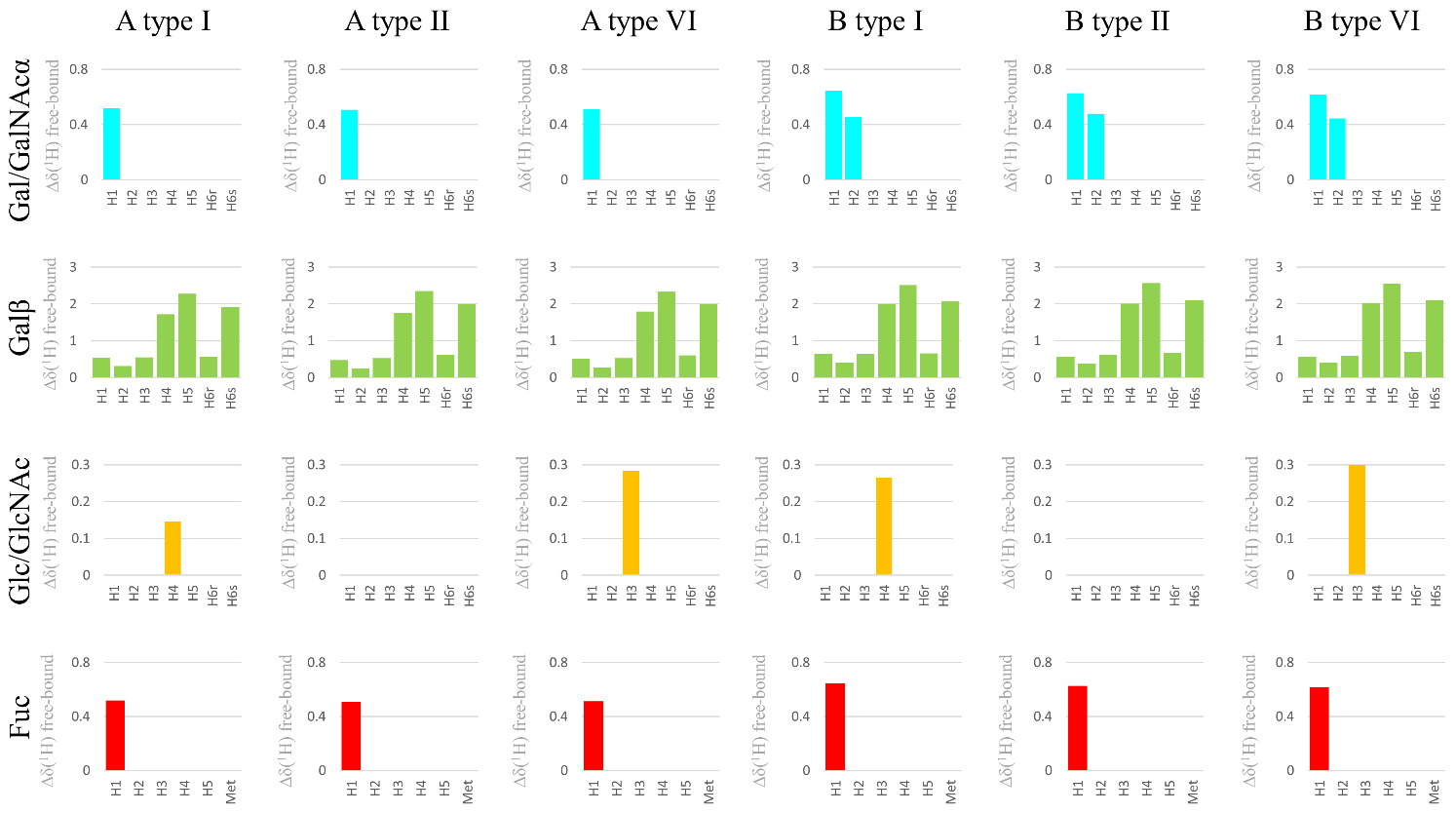


**Supplementary Figure S7.** Plot for the differences in chemical shifts between the free and bound states for every tetrasaccharide from trROESY experiments.

**Backbone Resonance Assignment**. The backbone resonance assignment of the N-terminal CRD of *h*Galectin-4 was performed at 25 °C on a 800 MHz Bruker spectrometer equipped with a cryoprobe. 3D HNCO, HN(CA)CO, HN(CO)CACB and HNCACB experiments were acquired and assigned for the free Gal-4N containing the His-Tag and for Gal-4N without the His-Tag in the presence of 200 equivalents of lactose. Additionally, HN(CO)CA and HNCA experiments were recorded for the free protein containing the His-Tag. The entire analysis provided the unambiguous identification of 80% of the expected NH signals for Gal-4N. The spectra were processed with Bruker TopSpin 3.5.2 and analysed via CARANMR 1.9.1.4.


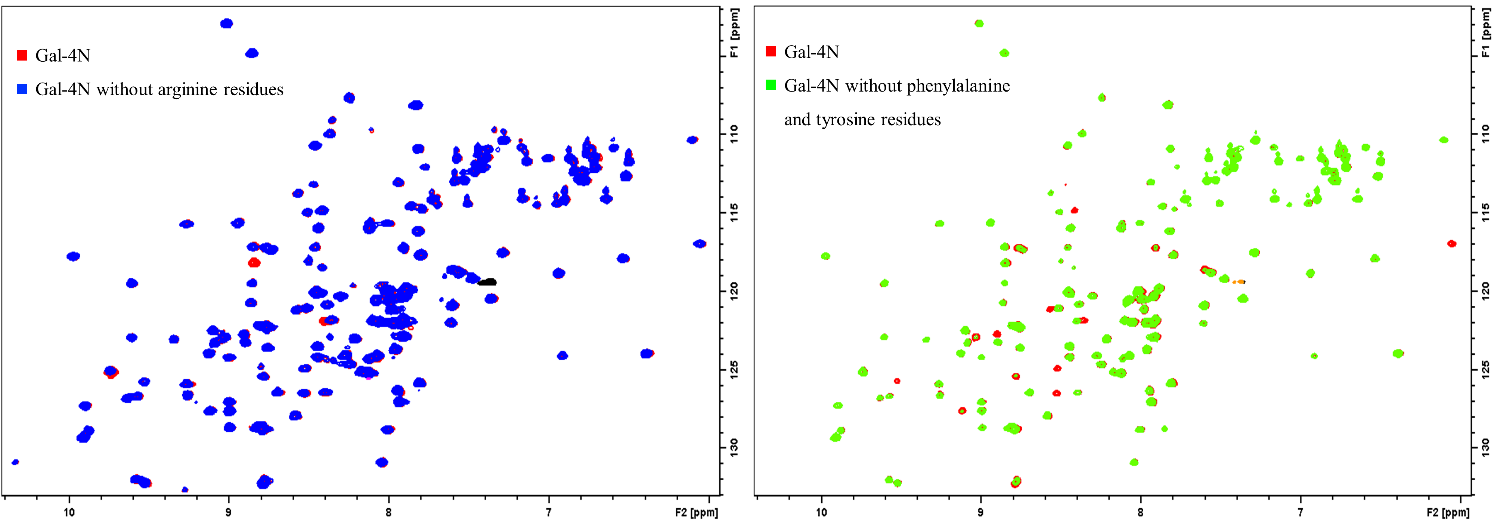
**Supplementary Figure S8.** Left: Superimposition of the ^1^H-^15^N HSQC spectra of Gal-4N (red) and Gal-4N with reduced peak intensity for arginine residues (blue). Right: Superimposition of the ^1^H-^15^N HSQC spectra of Gal-4N (red) and Gal-4N with reduced peak intensity for phenylalanine and tyrosine residues (green).


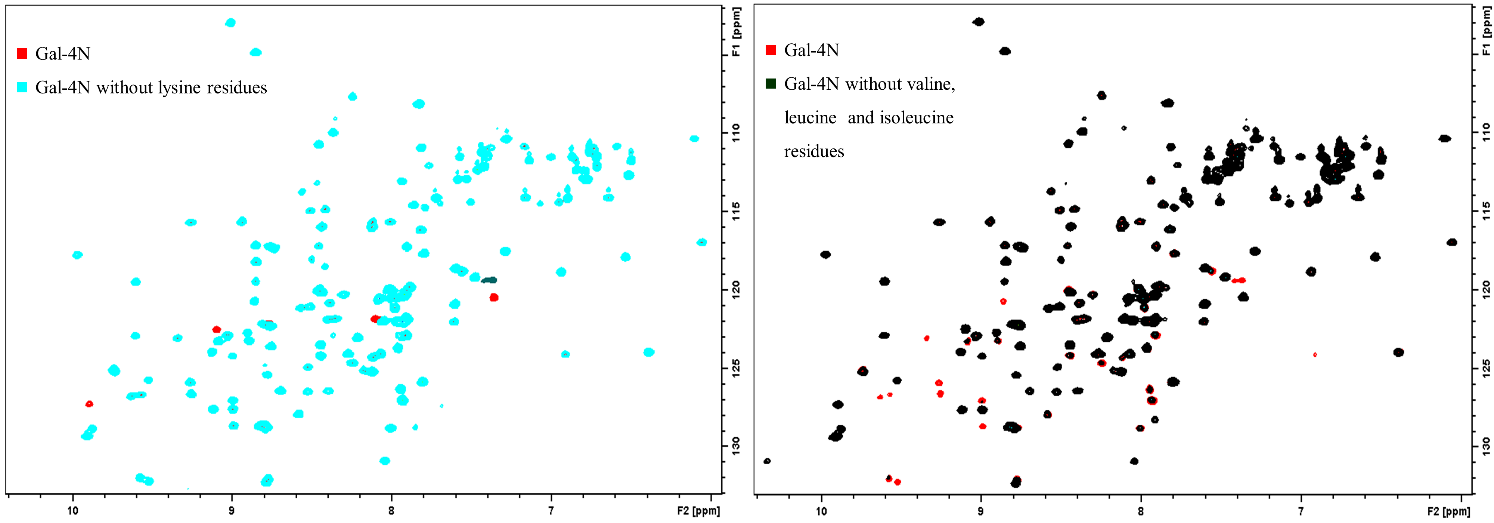


**Supplementary Figure S9.** Left: Superimposition of the ^1^H-^15^N HSQC spectra of Gal-4N (red) and Gal-4N with reduced peak intensity for lysine residues (light blue). Right: Superimposition of the ^1^H-^15^N HSQC spectra of Gal-4N (red) and Gal-4N with reduced peak intensity for valine, leucine and isoleucine residues (black).

**Chemical shift perturbations at Gal-4N**

The experiments were acquired using a 800 MHz Bruker spectrometer with a cryoprobe. The samples were prepared using 50 µM of the ^15^N labelled lectin in 50 mM sodium phosphate, 150 mM NaCl buffer (90:10 H_2_O:D_2_O). The experiments were acquired at 298 K. Six to nine points were recorded for each ligand and chemical shift perturbation (CSP) and dissociation constants were calculated using CcpNmr Analysis 2.4.2. Average ^1^H and ^15^N CSP was calculated for NH groups of the protein backbone using the formula: Δδ (ppm) = [(ΔδH^2^ + (0.14·ΔδN)^2^)/2]^½^) and the results were plotted in graphics.


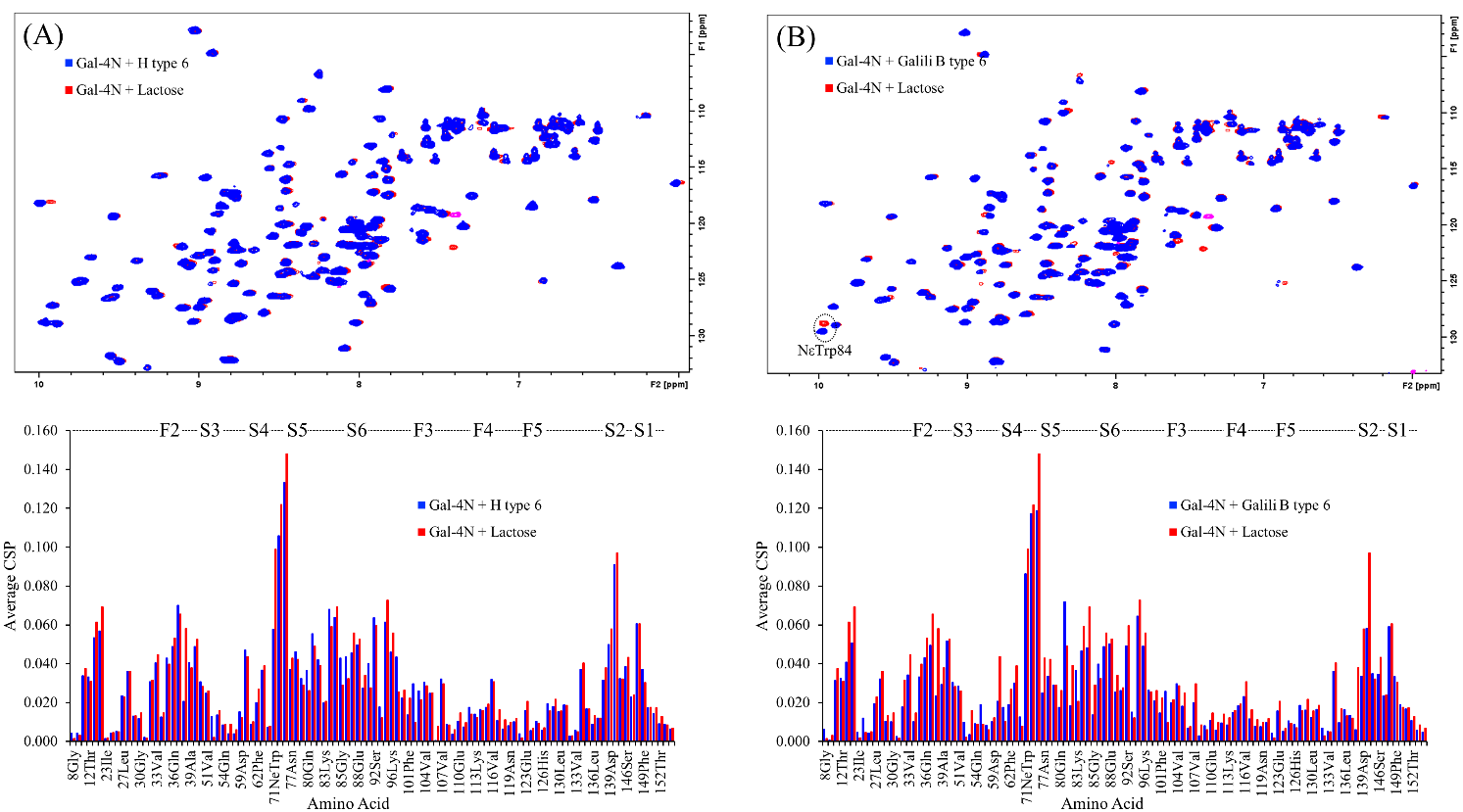


**Supplementary Figure S10.** A) Top: Superimposition of the ^1^H-^15^N HSQC spectra of Gal-4N saturated with H type-6 (blue) and lactose (red). Bottom: Chemical Shift Perturbation of Gal-4N with H type-6 (blue) and lactose (red). B) Top: Superimposition of the ^1^H-^15^N HSQC spectra of Gal-4N saturated with group B type-6 trisaccharide (blue) and lactose (red). Bottom: Chemical Shift Perturbation of Gal-4N with B type-6 trisaccharide (blue) and lactose (red).


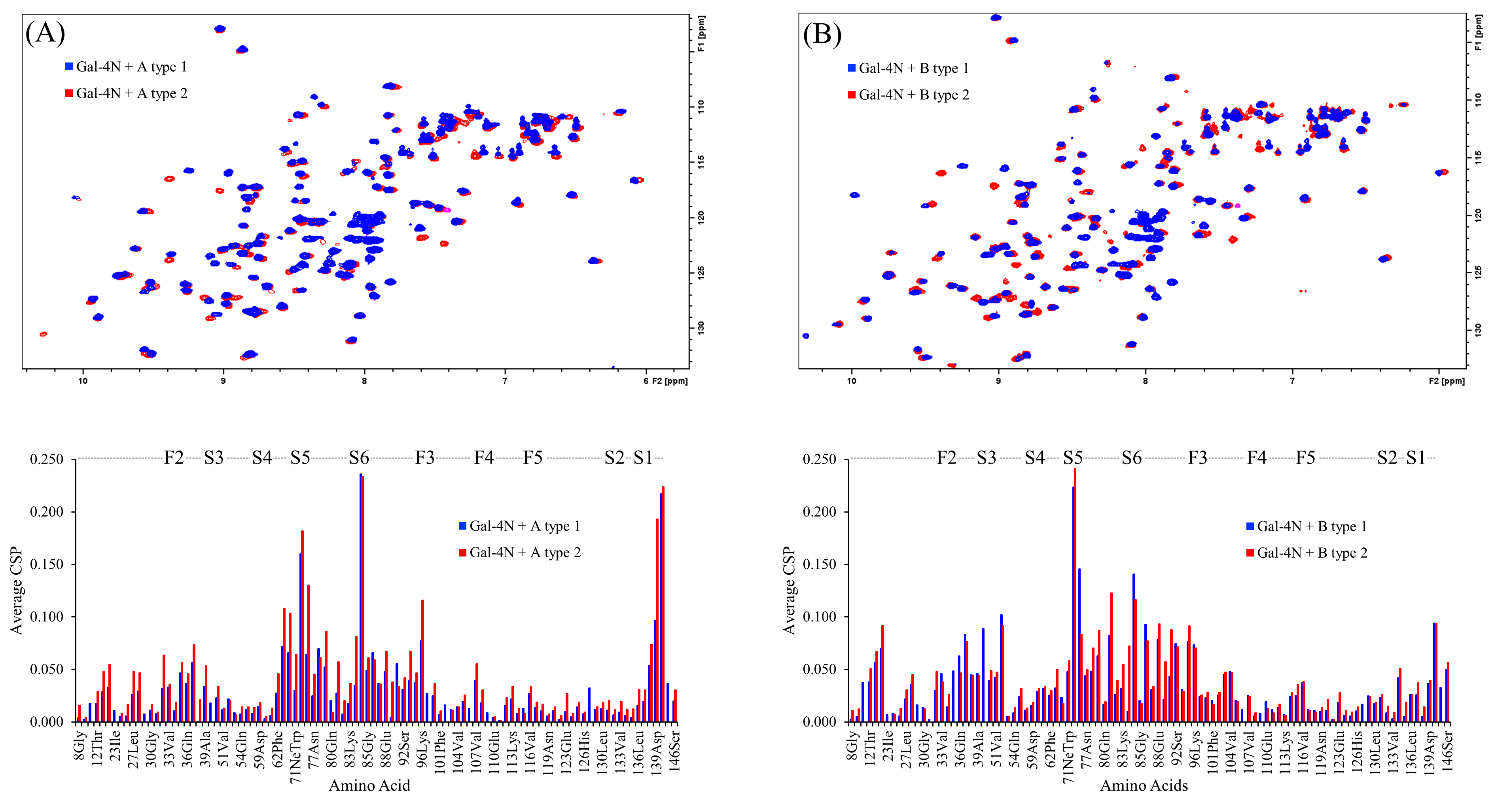


**Supplementary Figure S11.** A) Top: Superimposition of the ^1^H-^15^N HSQC spectra of Gal-4N saturated with A type-1 (blue) and A type-2 (red). Bottom: Chemical Shift Perturbation of Gal-4N with A type-1 (blue) and A type-2 (red). B) Top: Superimposition of the ^1^H-^15^N HSQC spectra of Gal-4N saturated with B type-1 (blue) and B type-2 (red). Bottom: Chemical Shift Perturbation of Gal-4N with B type-1 (blue) and B type-2 (red).


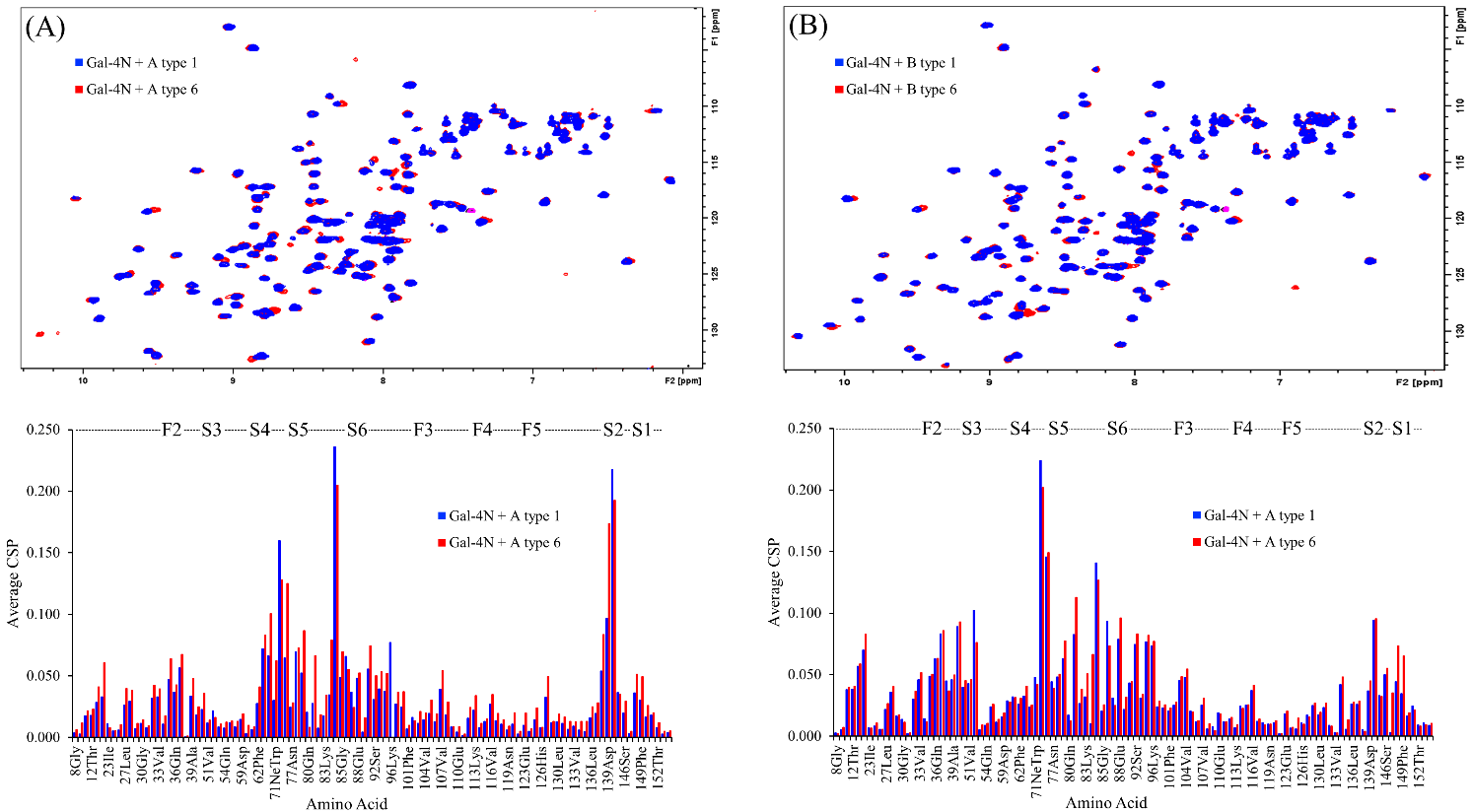


**Supplementary Figure S12.** A) Top: Superimposition of the ^1^H-^15^N HSQC spectra of Gal-4N saturated with A type-1 (blue) and A type-6 (red). Bottom: Chemical Shift Perturbation of Gal-4N with A type-1 (blue) and A type-6 (red). B) Top: Superimposition of the ^1^H-^15^N HSQC spectra of Gal-4N saturated with B type-1 (blue) and B type-6 (red). Bottom: Chemical Shift Perturbation of Gal-4N with B type-1 (blue) and B type-6 (red).


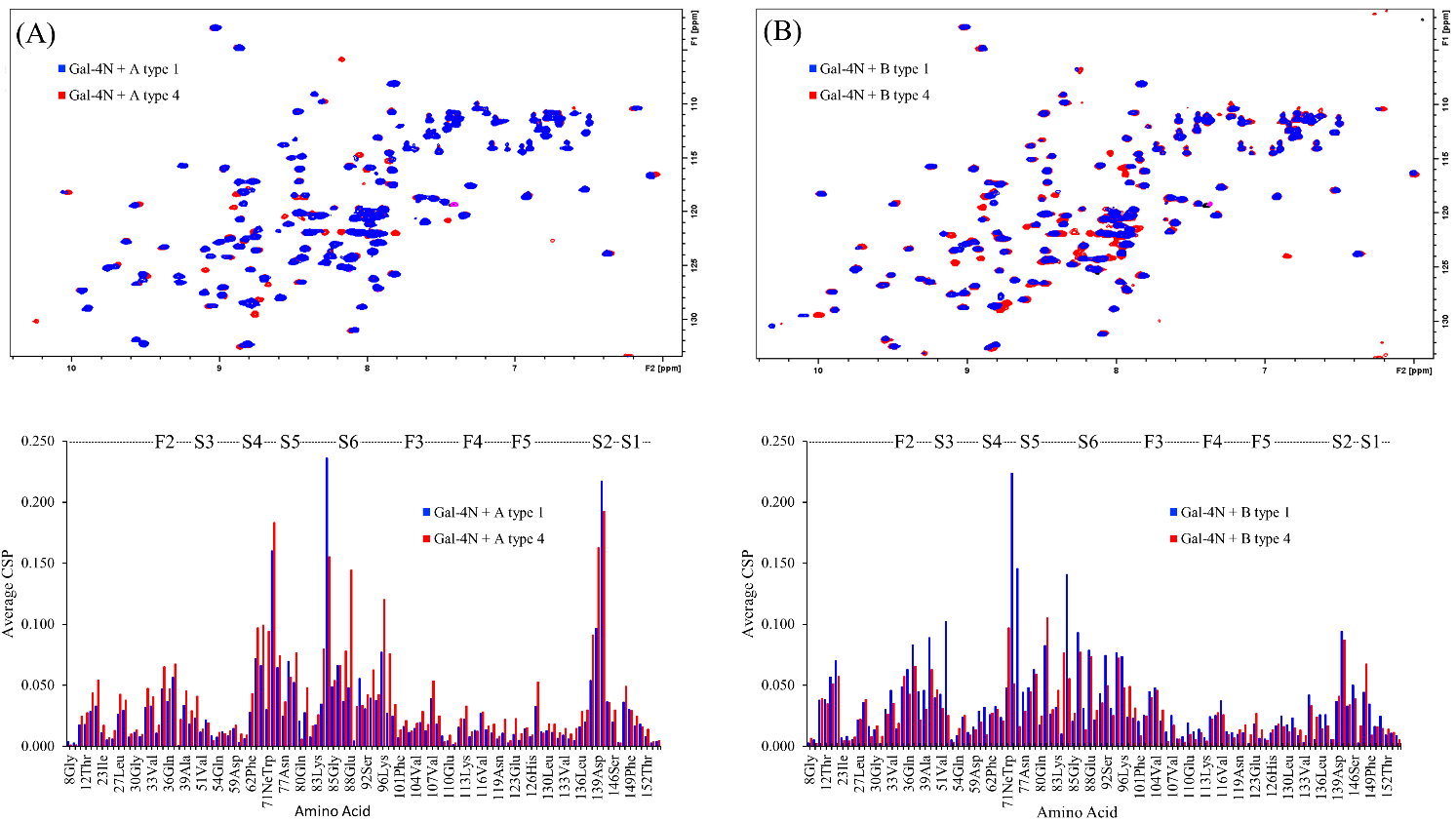


**Supplementary Figure S13.** A) Top: Superimposition of the ^1^H-^15^N HSQC spectra of Gal-4N saturated with A type-1 (blue) and A type-6 (red). Bottom: Chemical Shift Perturbation of Gal-4N with A type-1 (blue) and A type-6 (red). B) Top: Superimposition of the ^1^H-^15^N HSQC spectra of Gal-4N saturated with B type-1 (blue) and B type-6 (red). Bottom: Chemical Shift Perturbation of Gal-4N with B type-1 (blue) and B type-6 (red).

**Isothermal titratrion calorimetry (ITC)**. Isothermal Titration Calorimetry experiments were performed using MicroCal PEAQ-ITC calorimeter. Samples containing 100-200 µM of Gal-4N in PBS (50 mM sodium phosphate pH 7.4, 300 mM NaCl) were titrated with stocks of 3-10 mM in PBS of glycans A type 1, A type 2, A type 6, B type 1, B type 2 and B type 6. During the automated experiment, small aliquots (2-3 µL) of the sugar stocks were added to the cell containing the lectin. The association constants and the thermodynamic profile of the binding was calculated from the fitting of the titration data to a single binding site model.


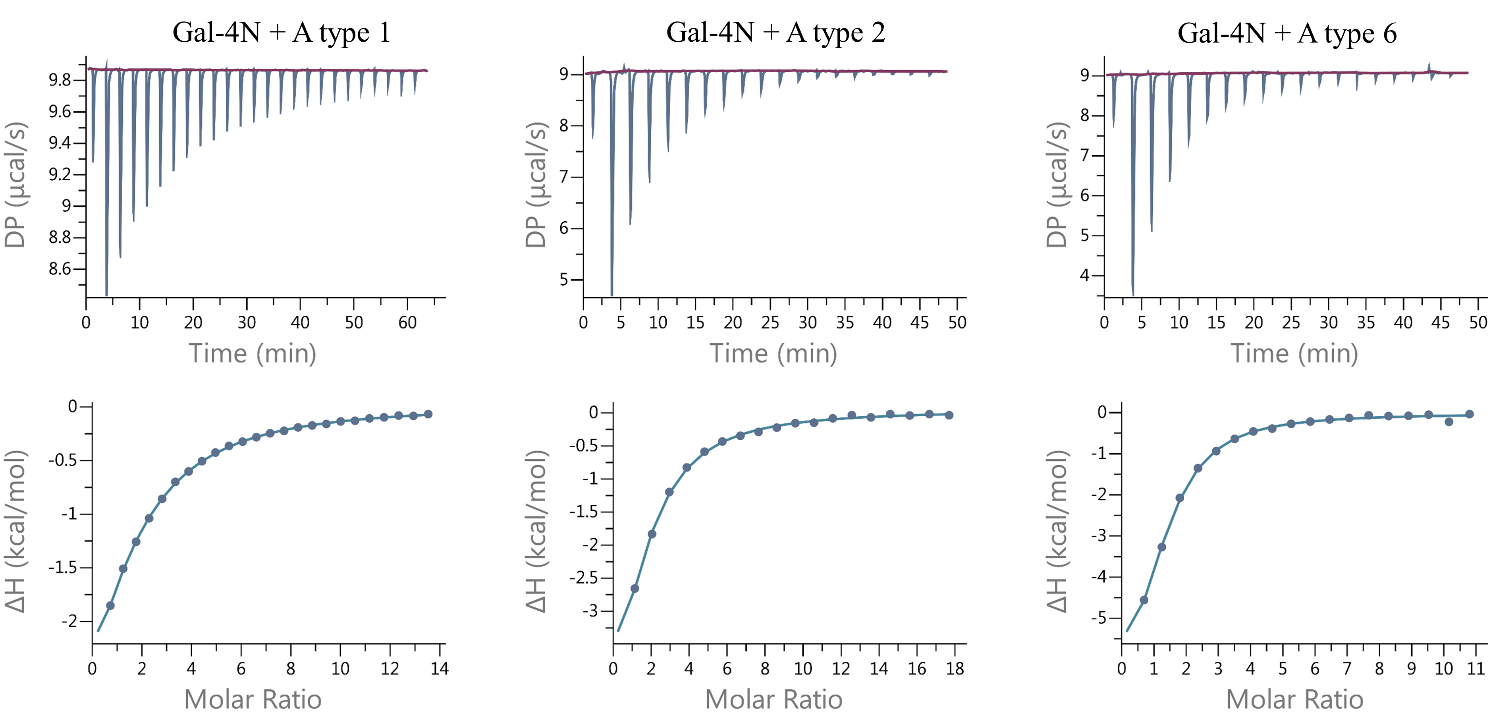


**Supplementary Figure S14.** Examples of titration profiles of titration of Gal-4N with group A tetrasaccharides. Top: Row data from the titration representing µcal/s dispersed during time. Bottom: The enthalpy of binding (kcal/mol) for each injection plotted against the protein/ligand molar ratio. The continuous line represents the least-squares-fit of the data to a single-site binding model.


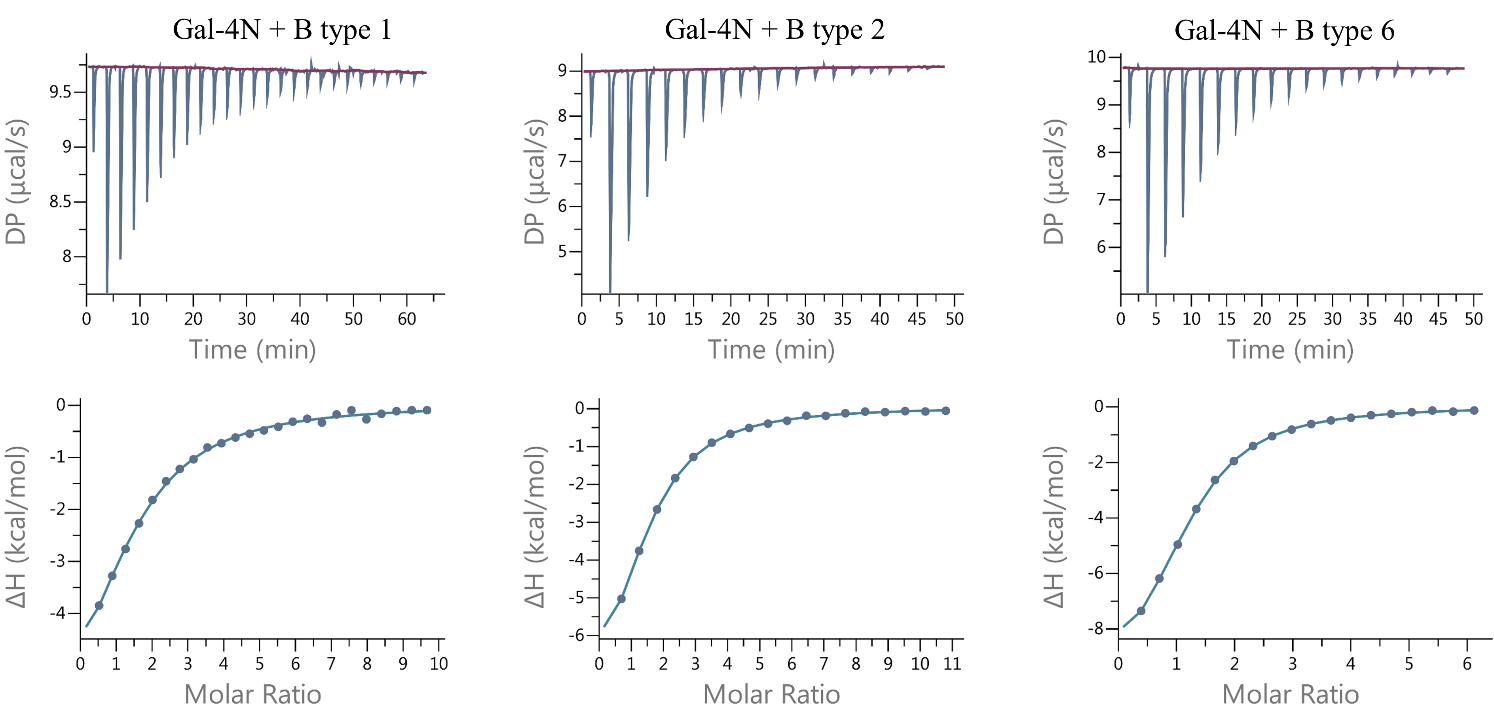


**Supplementary Figure S15.** Examples of titration profiles of titration of Gal-4N with group B tetrasaccharides. Top: Row data from the titration representing µcal/s dispersed during time. Bottom: The enthalpy of binding (kcal/mol) for each injection plotted against the protein/ligand molar ratio. The continuous line represents the least-squares-fit of the data to a single-site binding model.


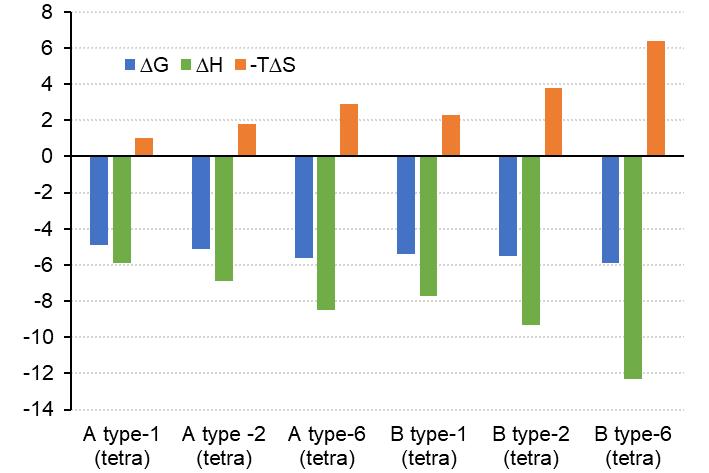


**Figure S16**. Binding free energies (Δ*G*), enthalpies (Δ*H*) and entropies (Δ*S*), in kcal mol^-1^, derived for ligands A/B-type-1/2/6 from Isothermal Titration Calorimetry (ITC) measurements.


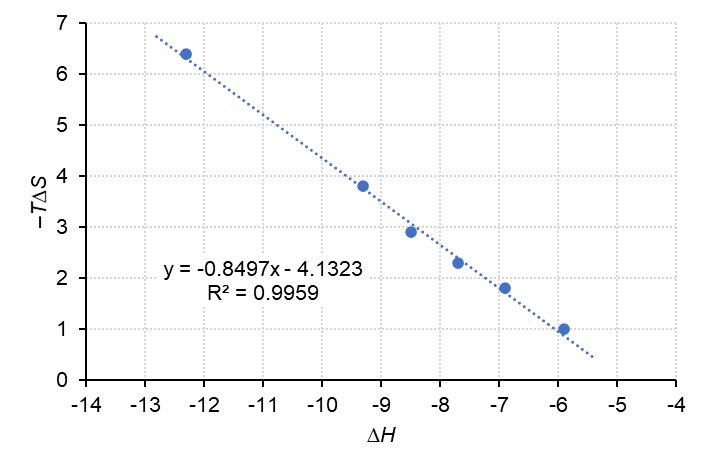


**Figure S17**. Binding enthalpies (Δ*H*) *versus* entropies (Δ*S*), in kcal mol^-1^, derived for ligands A/B-type-1/2/6 from Isothermal Titration Calorimetry (ITC) measurements. Note the nearly perfect linear relationship showing clear enthalpy/entropy compensation.
