# Supplementary figures and images for: Galectin-4 N-Terminal Domain: Binding Preferences Toward A and B Antigens With Different Peripheral Core Presentations
Source: Front Chem. 2021 Apr 21;9:664097. doi: 10.3389/fchem.2021.664097 (PMC8097242; doi:10.3389/fchem.2021.664097)

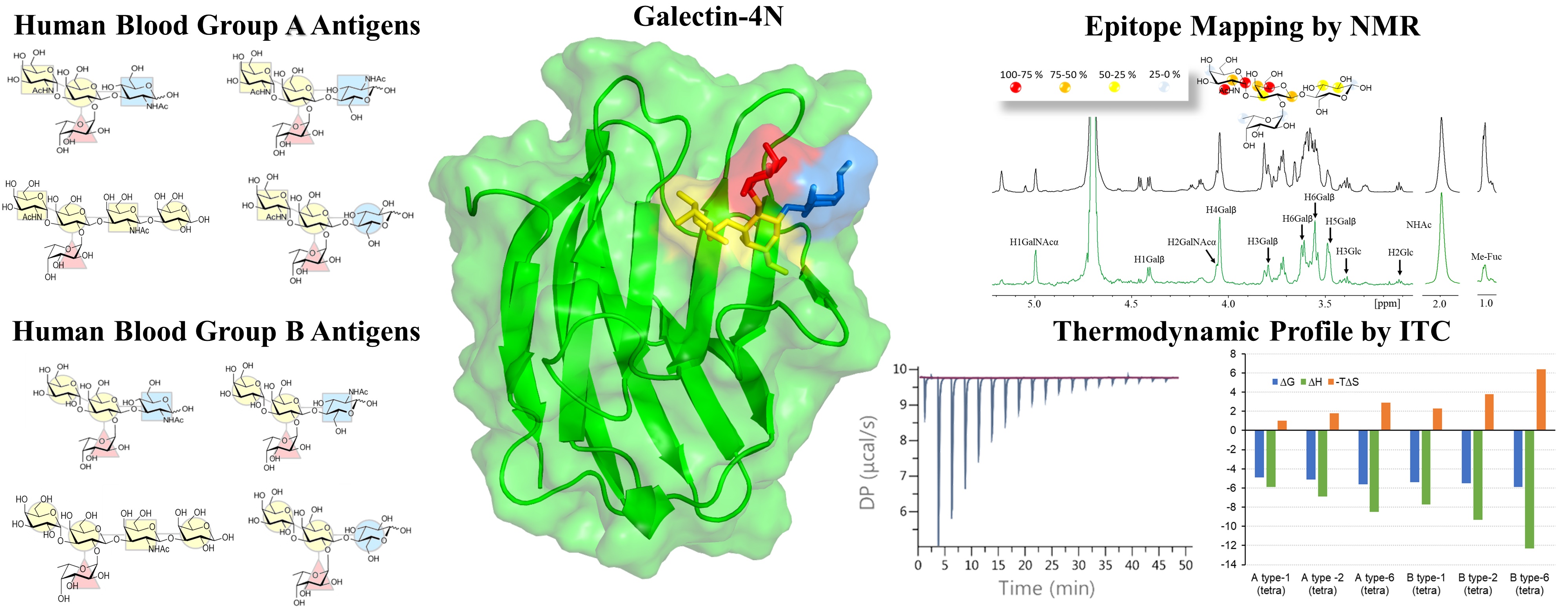

Supplement: Supplementary file 2 [file Image_1.JPEG]
